# The liver regulates ectopic calcification in *Abcc6*-deficient models of pseudoxanthoma elasticum

**DOI:** 10.1172/JCI193499

**Published:** 2026-03-10

**Authors:** Yijie Wang, Baiming Sun, Feiyang Ma, Bo Tao, Yiqian Gu, Zhiqiang Zhou, Jason Kim, Linlin Zhang, Zhihao Liu, Johanna ten Hoeve, Linsey Stiles, Lucia Fernandez del Rio, Calvin Pan, Orian Shirihai, Shili Xu, Thomas G. Graeber, Tamer Sallam, Matteo Pellegrini, Aldons J. Lusis, Arjun Deb

**Affiliations:** 1 Division of Cardiology, Department of Medicine, David Geffen School of Medicine,; 2 UCLA Cardiovascular Theme, David Geffen School of Medicine,; 3 Department of Molecular, Cell and Developmental Biology,; 4 Eli and Edythe Broad Center of Regenerative Medicine and Stem Cell Research,; 5 Molecular Biology Institute,; 6 California Nanosystems Institute,; 7 Bioinformatics Interdepartmental Program,; 8 Department of Microbiology, Immunology and Molecular Genetics,; 9 Department of Human Genetics, David Geffen School of Medicine,; 10 UCLA Metabolomics Center,; 11 Crump Institute of Molecular Imaging,; 12 Department of Molecular and Medical Pharmacology,; 13 Department of Medicine, Endocrinology, David Geffen School of Medicine,; 14 Jonsson Comprehensive Cancer Center, David Geffen School of Medicine, and; 15 Eli and Edythe Broad Center of Regenerative Medicine and Stem Cell Research, UCLA, Los Angeles, California, USA.

**Keywords:** Cardiology, Metabolism, Fibrosis, Genetic diseases

## Abstract

Pseudoxanthoma Elasticum (PXE) is a rare disease caused by loss of function of the ATP-binding cassette C (ABC) member 6 (*Abcc6*) gene and characterized by ectopic calcification of multiple tissues, but the physiological reasons underlying ectopic calcification in PXE remain unclear. In a murine model of *Abcc6*-deficient PXE in which animals developed robust cardiac calcification after heart injury, we show the critical importance of the liver in mediating ectopic cardiac calcification. Tissue-specific deletion of *Abcc6* in the liver, but not in the heart, was sufficient to cause post-injury cardiac calcification. Metabolomics and gene expression analysis demonstrated deficiencies in nucleotide metabolism, cellular energetics, and defects in cellular respiration underlying ectopic calcification in PXE. Functional abnormalities in cellular respiration in the injured heart were similar in animals with global or liver-specific *Abcc6* deficiency, showing that hepatic *Abcc6* expression regulated cellular respiration in the injured heart. We show that ectopic calcification in PXE was primarily dystrophic and that treatment with clodronate or etidronate, which prevent the growth of calcium hydroxyapatite mineralization, was sufficient to rescue the phenotype of ectopic cardiac calcification in *Abcc6*-deficient states. Taken together, these observations highlight the role of the liver in regulating target tissue metabolic and mitochondrial function in causing ectopic calcification in *Abcc6*-deficient states.

## Abstract 2

The liver regulates ectopic calcification of target tissues in the rare disease Pseudoxanthoma elasticum by altering the metabolome and cellular respiration of target organs.

## Introduction

Pseudoxanthoma elasticum (PXE) is a rare genetic disorder characterized by progressive ectopic calcification of the soft tissues and internal organs (1). PXE is inherited in an autosomal-recessive manner and affects between 1 of 25,000to 1 of 50,000 individuals and is caused by mutations in the ATP-binding cassette C (ABC) member 6 (*ABCC6*) gene on chromosome 16p13 (2–4). Ectopic calcification of soft tissues is the primary cause of mortality and morbidity in PXE and can affect blood vessels, eyes, skin and skeletal muscle (5, 6). Calcification of the coronary arteries or subendocardial/myocardial calcification in the heart can lead to premature cardiac disease or sudden death in affected individuals (7, 8). Ectopic calcification in affected individuals is commonly initiated by trauma or injury to the underlying organ, and individuals are often in early adulthood when devastating clinical complications of ectopic calcification can appear including loss of vision secondary to ectopic calcification in the eye (9). The mechanisms by which loss of function of *Abcc6* leads to ectopic calcification of tissues are not understood, and there are currently no therapies for retarding progressive calcification.

*Abcc6* is thought to be a transporter, but the identity of the molecule it transports has remained a mystery (10, 11). Circulating calcium and phosphate levels are usually within normal levels in individuals with PXE (12). The circulating levels of the mineralization inhibitor, pyrophosphate (PPi) have been noted to be decreased in individuals with PXE, but a clear correlation with PPi and phenotypes in individuals with PXE is lacking (13, 14). Whether loss of local tissue expression of the gene creates a permissive environment for mineralization to occur or whether *Abcc6* acts in a systemic manner to promote mineralization of tissues is also not clear.

Murine models of *Abcc6* deficiency have been generated, and calcification of whiskers in these animals demonstrated the propensity of these animals to recapitulate in part the human calcific phenotype (15). However, calcification of the skin and whiskers takes months, which makes it in part difficult to discern mechanisms of *Abcc6*-mediated calcification. In this body of work, we create a robust model of cardiac calcification in *Abcc6-*deficient animals (11, 16–18) by inducing cardiac cryo-injury and demonstrate extensive deposition of calcium mineralization in the heart within 72 hours of injury. Using this model, we shed light on the pathogenesis of ectopic calcification in PXE. We show that *Abcc6* acts in a noncardiac, cell-autonomous manner to cause cardiac calcification. The deletion of *Abcc6* in the liver was sufficient to cause cardiac calcification, while the deletion of *Abcc6* in cardiac muscle did not cause cardiac calcification. We show that the deletion of *Abcc6* in the liver led to widespread metabolic abnormalities in the injured heart, affecting purine, pyrimidine, and NAD metabolism with severe decreases in cellular respiration in isolated mitochondria. These findings demonstrate that hepatic *Abcc6* expression regulates cardiac mitochondrial function after injury. Calcification in the cardiovascular system is regarded as having parallels with osteogenesis, in which the calcium deposits in cardiovascular tissues occur from mesenchymal cells, adopting an osteogenic transcription program. We show that the rapid calcification of the heart in *Abcc6* deficient animals after cardiac injury was initiated within the cardiac muscle cell and occurred in a dystrophic manner without any evidence of active osteogenesis. Bisphosphonates are compounds that are known to bind to calcium hydroxyapatite and prevent further mineralization growth (19). In animals deficient in *Abcc6*, we show that injection of the bisphosphonates clodronate or etidronate led to complete rescue of the calcific phenotype.

## Results

### Animals globally deficient in Abcc6 develop rapid and robust cardiac calcification after heart injury.

**Figure 1:**
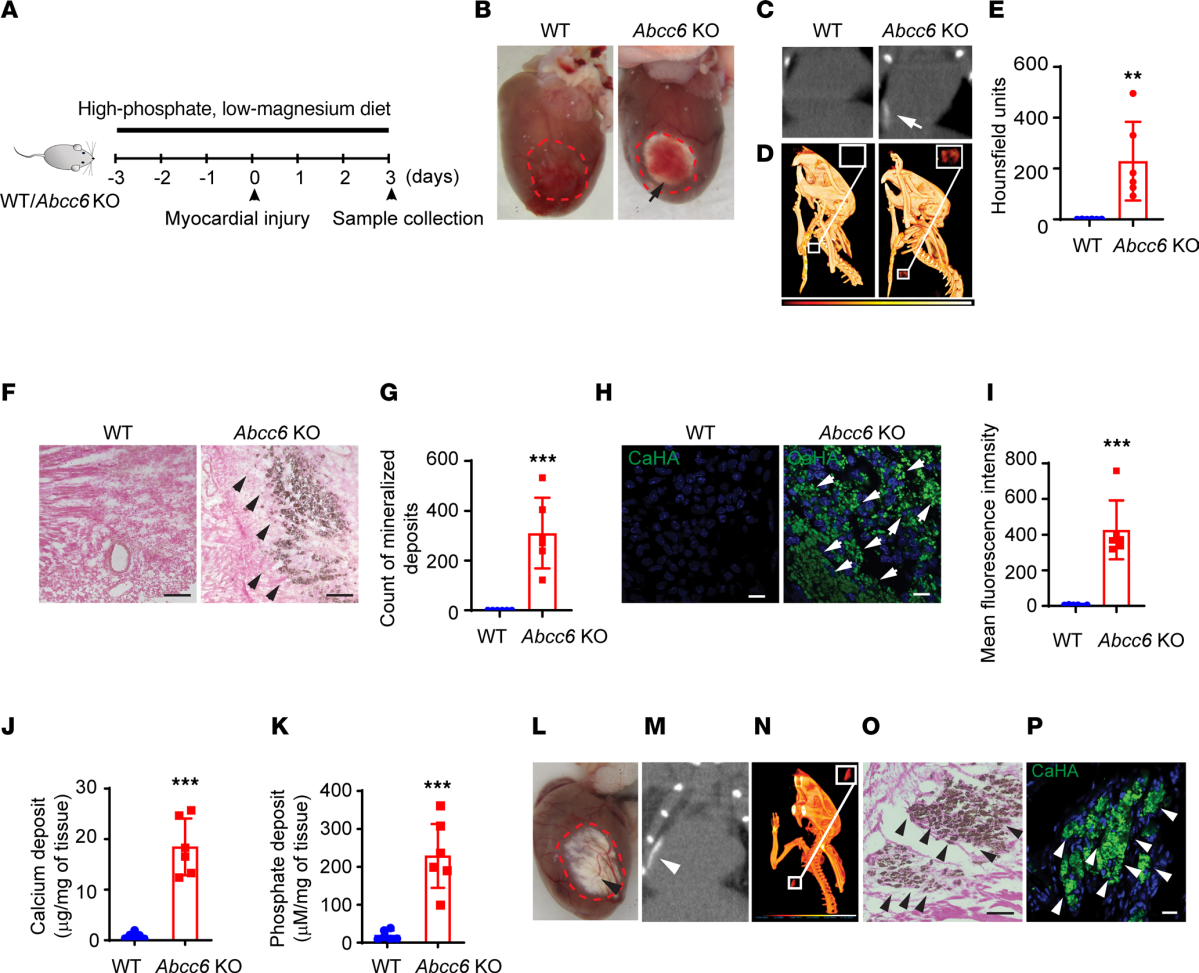
Genetic deletion of *Abcc6* leads to persistent myocardial calcification after heart injury. (**A**) Schematic of the experimental design with cryo-induced injury of WT or *Abcc6*-KO animals and harvesting 3 days after injury. A high-phosphate, low-magnesium diet was administered starting 3 days before surgery. (**B**) Cryo-injured WT and *Abcc6*-KO animals demonstrating cardiac calcification (black arrow) on gross inspection and calcification in the *Abcc6*-KO heart, visualized with (**C**) CT scan and (**D**) following 3D rendering (red circles and insets indicate the calcific lesions). (**E**) Quantitative analysis of calcium content in scar tissue as measured by CT imaging (*n* = 6 in each group, mean ± SD, **P < 0.01, by 2-tailed, unpaired *t* test, WT vs. *Abcc6*-KO group). (**F** and **G**) Histological staining of cryo-injured myocardium in WT and *Abcc6*-KO animals with (**F**) Von-Kossa staining (scale bars: 100 μm) and (**G**) corresponding quantitative analysis showing calcium deposits (black arrows) (*n* = 6 in each group, mean ± SD, ***P < 0.001, by 2-tailed, unpaired *t* test, WT vs. *Abcc6*-KO group). (**H**) Immunostaining for hydroxyapatite (white arrows, scale bars: 10 μm) and (**I**) corresponding quantitative analysis. (*n* = 6 in each group, mean ± SD, ***P < 0.001 WT versus *Abcc6*-KO group, *P* value was calculated by 2-tailed, unpaired *t* test). (**J** and **K**) Biochemical measurements of (**J**) myocardial calcium and (**K**) phosphate deposits. All staining and measurements were performed on day 3 following injury (*n* = 6 in each group, mean ± SD, ***P < 0.001, by 2-tailed, unpaired *t* test, WT vs. *Abcc6*-KO group). (**L–P**) Persistence of cardiac calcification with (**L**) a gross image of the heart 4 months after cryo-injury (black arrowhead indicates the calcific lesion), (**M**) CT scan (white arrowhead), and (**N**) 3D rendering showing the calcification region. Red circles (**L**) and insets (**N**) indicate the calcific lesions. (**O** and **P**) Histological analysis of cryo-injured myocardium 4 months after surgery. (**O**) Von-Kossa staining (scale bar: 100 μm) and (**P**) immunostaining for hydroxyapatite (black and white arrowheads; scale bar: 50 μm). (*n* = 6 per group; WT vs. *Abcc6*-KO).

To investigate the underlying mechanisms of ectopic calcification in PXE, we created a model of robust cardiac calcification that occurs rapidly in *Abcc6*-deficient animals (11). We subjected animals that were globally deficient in *Abcc6* (*Abcc6* KO) to a phosphate-rich, magnesium-deficient diet for 3 days prior to and 3 days following cardiac cryo-injury (Figure 1A). Following cryo-injury to the left ventricle, gross inspection revealed extensive calcification in the injured cardiac region of *Abcc6*-KO animals 3 days after injury, whereas the WT hearts exhibited only pale tissue at the injury site without any evidence of mineralization (Figure 1B). Micro-CT imaging with 3D rendering of the heart and thoracic cage confirmed the presence of cardiac calcification in *Abcc6-*KO animals but not in WT controls (Figure 1, C–E). Von-Kossa staining of tissue sections confirmed the presence of calcium deposits in the injured left ventricle of *Abcc6*-KO animals (Figure 1, F and G). Immunofluorescence staining with dyes that bind to hydroxyapatite confirmed the mineral deposits to be hydroxyapatite (Figure 1, H and I). Biochemical measurement of calcium and phosphate in tissue homogenates of injured heart confirmed increased calcium and phosphate deposition in *Abcc6*-KO animals (0.8894 ± 0.6156 vs. 18.49 ± 5.619 μg/mg tissue of Ca^2+^ and 19.43 ± 12.75 vs. 228.7 ± 84.27 μM/mg tissue of phosphate in WT and *Abcc6*-KO animals, respectively, *P* < 0.001, mean ± SD) (Figure 1, J and K). The serum calcium, phosphorus, and magnesium levels were not significantly different between WT and the *Abcc6*-KO animals (Supplemental Figure 1; supplemental material available online with this article; https://doi.org/10.1172/JCI193499DS1). TTC staining of the harvested heart 3 days following injury demonstrated similar degrees of dead and viable myocardium in WT and *Abcc6*-KO hearts (Supplemental Figure 2, A and B), and the number of accumulated fibroblasts in the injured region was not different between the WT and the *Abcc6*-KO animals (Supplemental Figure 2, C and D), suggesting that differences in myocyte viability did not affect the calcific response. The calcification persisted beyond the acute phase of injury and remained visible at 4 months after cardiac cryo-injury (Figure 1, L–P).

### The immune system probably does not contribute to calcification in Abcc6-deficient animals.

To gain insight into the transcriptional changes that are associated with such rapid calcification of the injured heart, we performed bulk RNA-seq of injured calcific tissue of the *Abcc6-*KO animal versus the injured noncalcific tissue of the WT animal at day 3 of cryo-injury. Gene ontogeny analysis demonstrated that the principal pathways differentially upregulated in calcific tissue of *Abcc6-*KO hearts related to extracellular matrix (ECM) and inflammation (Supplemental Figure 3A). Genes downregulated in calcific tissue were related to cardiac muscle contraction and myofibril assembly (Supplemental Figure 3B). Inflammation has been strongly related to calcification of cardiovascular tissues (20, 21).

Given the profound differences in inflammation and ECM gene expression, we performed single-cell transcriptomics to obtain greater insight into cell-specific transcriptomics including differences in the inflammatory infiltrate that could create a more permissible environment for calcification. The cell population was clustered into typical cellular identities on the basis of expression of canonical genes in specific cell types (Supplemental Figure 4), and we could discern multiple populations of cells including cardiac muscle cells, endothelial cells, fibroblasts, macrophages, and other immune cell subsets (Supplemental Figure 3C). We found that cell populations were equally distributed between the *Abcc6*-KO and WT genotypes with the exception of macrophages, which showed distinct subsets between the WT and the *Abcc6*-KO injured hearts (Supplemental Figure 3D). The macrophage population could be separated into distinct subclusters (Supplemental Figure 3E) according to gene expression profile, and clusters 1, 3, and 4 predominated more in the tissue of *Abcc6*-KO hearts (Supplemental Figure 3F), and the macrophage population enriched in the calcific tissue of *Abcc6*-KO animals represented both monocyte-derived macrophages and tissue repair macrophages (M2) (Supplemental Figure 3G). However, the fraction of macrophages recruited to the injured hearts of WT and *Abcc6*-KO mice was similar, as shown by both flow cytometry (Supplemental Figure 5, A and B) and immunofluorescence staining (Supplemental Figure 5, C and D)

**Figure 2:**
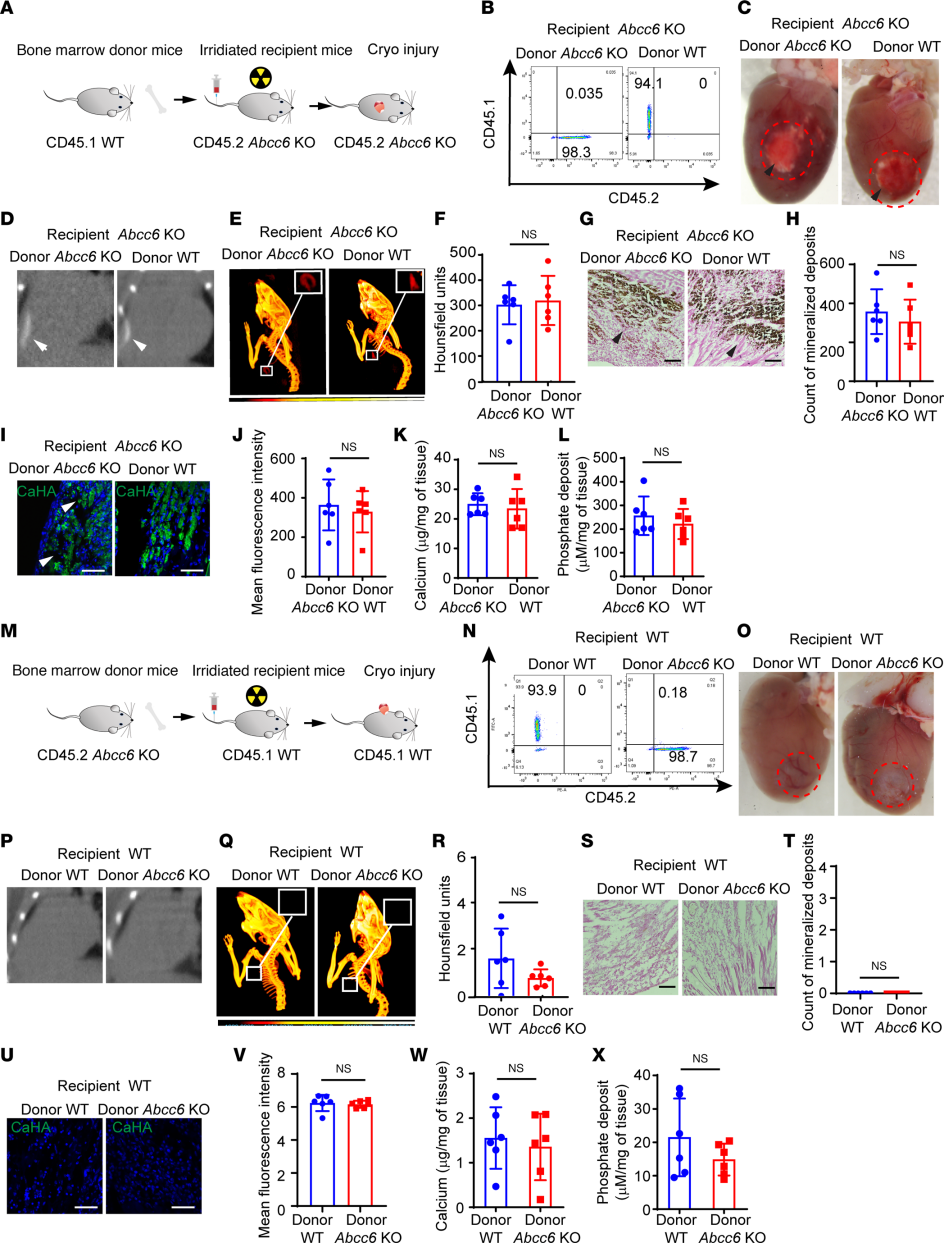
Bone marrow–derived cells do not affect ectopic cardiac calcification. (**A**) Schematic of BMT. CD45.2 *Abcc6*-KO recipients were irradiated and transplanted with bone marrow from CD45.1 WT or CD45.2 *Abcc6*-KO donors, followed by chimerism assessment at 8 weeks and cryo-injury at 9 weeks. (**B**) Analysis of chimerism in peripheral blood of CD45.2 recipients (*n* = 6 per group). (**C**) Gross images of hearts 3 days after cryo-injury in *Abcc6*-KO recipients; red dotted lines indicate injury; arrowheads indicate calcific lesions. (**D**) CT scan and (**E**) 3D rendering highlighting calcific lesions. (**F**) Quantitative analysis of calcification in scar tissue from CT data (*n* = 6 per group; mean ± SD; 2-tailed, unpaired Student’s *t* test) (**G** and **H**) Histological assessment with (**G**) Von-Kossa staining and (**H**) quantification. Scale bars: 100 μm (*n* = 6 per group; mean ± SD; 2-tailed, unpaired Student’s *t* test). (**I**) Immunostaining for hydroxyapatite and (**J**) quantification. Scale bars: 50 μm. (*n* = 6 per group; mean ± SD; 2-tailed, unpaired Student’s *t* test). (**K** and **L**) Biochemical measurements of myocardial (**K**) calcium and (**L**) phosphate (*n* = 6 per group; mean ± SD; 2-tailed, unpaired Student’s *t* test). (**M**) Schematic of BMT in CD45.1 WT recipients transplanted with CD45.2 *Abcc6*-KO marrow, followed by chimerism assessment and cryo-injury. (**N**) Chimerism analysis of peripheral blood from CD45.1 recipients (*n* = 6 per group). (**O**) Gross images of hearts 3 days after cryo-injury showing an absence of calcification, consistent with observations from (**P**) CT scans and (**Q**) 3D rendering. (**R**) Quantitative analysis of calcium content in scar tissue. (**S**) Von-Kossa staining and (**T**) quantification. Scale bars: 100 μm. (**U**) Immunostaining for hydroxyapatite and (**V**) quantification. Scale bars: 50 μm. (**W** and **X**) Biochemical measurements of myocardial (**W**) calcium and (**X**) phosphate (*n* = 6 per group; mean ± SD; 2-tailed, unpaired Student’s *t* test).

As the accumulation of different subsets of macrophages could be secondary to the mineralization itself rather than being causal, we next investigated, using bone marrow transplantation (BMT) experiments, whether the inflammatory cells played a critical causal role in mediating calcification in *Abcc6*-deficient states. For this purpose, we transplanted bone marrow from CD45.1 WT animals into irradiated CD45.2 *Abcc6*-KO mice (Figure 2A). We used an additional set of controls, in which *Abcc6*-KO bone marrow was transplanted into irradiated *Abcc6*-deficient animals. Eight weeks after transplantation, we performed chimerism analysis, and then 1 week later subjected the animals to cardiac cryo-injury to determine the effects on cardiac calcification (Figure 2A). Analysis of peripheral blood cells 8 weeks after BMT demonstrated that 96.5 ± 1.258 (mean ± SD) cells in the *Abcc6*-KO animals were of WT origin, while *Abcc6*-deficient animals that received *Abcc6*-deficient bone marrow cells did not exhibit detectable CD45.1^+^ cells (Figure 2B). We then subjected the *Abcc6*-KO animals that had been transplanted with WT bone marrow to cardiac cryo-injury. Despite successful chimerism, the *Abcc6*-KO animals transplanted with WT bone marrow cells developed myocardial calcification, as did those transplanted with *Abcc6*-KO bone marrow (Figure 2C). CT imaging demonstrated the presence of cardiac calcification in *Abcc6*-KO recipient animals transplanted with either WT or *Abcc6*-KO bone marrow (Figure 2, D–F). Consistent with these imaging findings, Von-Kossa staining of injured cardiac tissue (Figure 2, G and H), and immunofluorescence labeling of hydroxyapatite (Figure 2, I and J) confirmed that both groups developed comparable mineral deposits. Biochemical analysis of the calcified tissue showed similar degrees of calcium and phosphate deposition in both groups (Figure 2, K and L).

We next performed another series of BMT experiments to determine whether the presence of *Abcc6*-KO bone marrow cells would be sufficient to cause calcification. For this purpose, we irradiated and transplanted CD45.1 WT animals with donor *Abcc6*-KO bone marrow (CD45.2) (Figure 2M). Following successful engraftment, we subjected the recipient WT animals to cardiac cryo-injury (Figure 2M). We observed successful chimerism of donor *Abcc6*-KO cells in WT animals (Figure 2N). Analysis of hearts following cardiac cryo-injury demonstrated the absence of calcification in WT animals transplanted with *Abcc6*-KO bone marrow as well as in those transplanted with WT bone marrow (Figure 2O). This absence of calcification was further confirmed by micro-CT imaging (Figure 2, P–R), Von-Kossa staining (Figure 2, S and T), and hydroxyapatite immunofluorescence staining (Figure 2, U and V). Biochemical analysis demonstrated the absence of mineralization (Figure 2, W and X). Taken together, these experiments demonstrate that bone marrow–derived cells did not likely to contribute to the calcific phenotype in *Abcc6*-deficient states.

To further investigate the role of macrophages, we performed experiments to either block or deplete macrophage activity to confirm the phenotype seen in the BMT experiments. We subjected *Abcc6*-KO animals to a low-magnesium, high-phosphate diet as described above and subjected the animals to cardiac cryo-injury. We administered a monoclonal antibody (mAb) against the CSF1R or IgG control at 3 days and 1 day prior to injury and administered another dose 1 day after injury (Supplemental Figure 6A). Previous studies have demonstrated that injection of the CSF1R antibody depleted macrophages (22, 23), and flow cytometry of the injured heart harvested at 3 days after cryo-injury showed a significant reduction of macrophages in the injured region in the CSF1R mAb–injected animals (Supplemental Figure 6B). However, macrophage inhibition via administration of the CSF1R mAb did not have an effect on calcification. On gross inspection, we observed similar degrees of calcification between the IgG- and CSF1R mAb–injected groups (Supplemental Figure 6C). CT scanning revealed that CSF1R mAb treatment in *Abcc6*-KO mice did not result in any difference in cardiac tissue mineralization (Supplemental Figure 6, D–F). Histopathological staining and immunofluorescence detection of hydroxyapatite confirmed the inability of CSF1R mAb to affect calcification compared with IgG-injected controls (Supplemental Figure 6, G–J). Biochemical analysis of the calcified tissue revealed a similar amount of calcium and phosphate deposition in the IgG- and CSF1R mAb–treated groups (Supplemental Figure 6, K and L). Next, we performed a circulating monocyte/macrophage depletion experiment. For this purpose, we crossed transgenic mice expressing the diphtheria toxin receptor (DTR) under the *CD11b* promoter (*CD11b-DTR*), which enables selective depletion of CD11b cells, with the *Abcc6*-KO animals to create CD11b-DTR *Abcc6-*KO animals. The CD11b-DTR animals express the diphtheria toxin (DT) receptor under the control of the human CD11b promoter; thus, administration of DT would lead to ablation of CD11b circulating monocyte/macrophage lineages (24, 25). Macrophage depletion was achieved by 2 i.p. injections of DT (15 ng/g body weight) to ablate CD11b-expressing cells at 3 days and 1 day before injury (Supplemental Figure 6M). Using flow cytometry, we observed decreased macrophages in the hearts of animals that received DT (Supplemental Figure 6N), but on gross inspection, no difference in calcification was noted between the animals that had macrophages ablated and the control animals (Supplemental Figure 6O). CT scanning, histological staining, and immunofluorescence labeling of hydroxyapatite confirmed a similar degree of calcium deposition between the CD11b-ablated animals and the controls (Supplemental Figure 6, P–V). Consistent with these findings, biochemical analyses showed similar levels of calcium and phosphate in the injured cardiac tissue from animals of both groups (Supplemental Figure 6, W and X). Collectively, these experiments using BMT and macrophage depletion techniques strongly indicated that immune cells did not directly affect the degree of calcification in *Abcc6*-KO animals after cardiac cryo-injury.

### Deficiency of Abcc6 in the liver but not in the heart regulates ectopic calcification in the heart.

Given that bone marrow–derived cells do not contribute to ectopic calcification, we next investigated how the expression of *Abcc6* in the heart or other tissues affects the calcific phenotype. To investigate this, we first analyzed *Abcc6* expression across various organs using the Genotype-Tissue Expression (GTEx) database. Our analysis revealed that *Abcc6* was most highly expressed in the liver, whereas cardiac tissue had minimal expression (Supplemental Figure 7).

**Figure 3:**
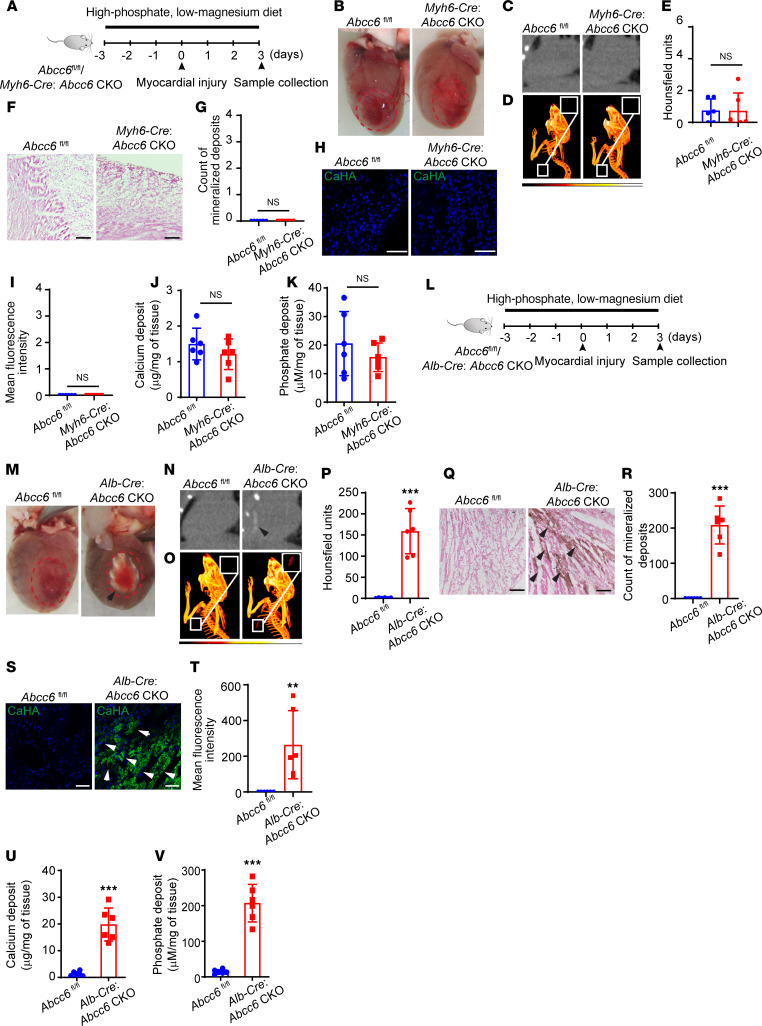
Liver- but not cardiac muscle–specific deletion of *Abcc6* leads to ectopic calcification following heart injury. (**A**) Experimental design of cryo-injury in *Myh6-Cre*: *Abcc6*-CKO animals on a high-phosphate, low-magnesium diet. (**B**) Gross inspection of the cryo-injured hearts from *Abcc6^fl/fl^* and *Myh6-Cre* *Abcc6*-CKO mice 3 days after injury. (**C**) CT scan, (**D**) corresponding 3D rendering, and (**E**) quantitative analysis of calcium contents in scar tissue demonstrating a lack of detectable calcification (*n* = 6 per group; mean ± SD; 2-tailed, unpaired *t* test). (**F** and **G**) Histological staining of cryo-injured myocardium in WT and *Abcc6*-KO animals with (**F**) Von-Kossa staining and (**G**) corresponding quantitative analysis showing calcium deposits (*n* = 6 per group; mean ± SD; 2-tailed, unpaired *t* test). (**H**) immunostaining for hydroxyapatite and (**I**) corresponding quantitative analysis (*n* = 6 per group; mean ± SD; 2-tailed, unpaired *t* test). (**J** and **K**) Biochemical measurements of (**J**) myocardial calcium (**K**) and phosphate deposits in the injured region (*n* = 6 per group; mean ± SD; 2-tailed, unpaired *t* test). (**L**) Experimental design of inducing cryo-injury in animals with liver-specific deletion of *Abcc6*. (**M**) Gross inspection of cryo-injured hearts from *Abcc6^fl/fl^* and *Alb-Cre* *Abcc6*-CKO animals 3 days after injury. Red dashed circles indicate the injury region with visible calcification in animals with liver-specific *Abcc6* deletion. Black arrowhead indicates the calcification. (**N**) CT scan, (**O**) 3D rendering, and (**P**) corresponding quantitative analysis of calcium content in scar tissue showing calcification in the injured hearts of liver-specific *Abcc6*-deleted animals (*n* = 6 per group; mean ± SD; ***P < 0.001, by 2-tailed, unpaired *t* test). (**Q** and **R**) Myocardial calcification region with (**Q**) Von-Kossa staining and (**R**) the corresponding quantitative analysis (*n* = 6 per group; mean ± SD; ***P < 0.001, by 2-tailed, unpaired *t* test). (**S**) Immunostaining for hydroxyapatite in injured hearts of *Abcc6^fl/fl^* and *Alb-Cre*: *Abcc6*-CKO mice and (**T**) the corresponding quantitative analysis (*n* = 6 per group; mean ± SD; **P < 0.01, by 2-tailed, unpaired *t* test). (**U** and **V**) Biochemical measurements of (**U**) myocardial calcium and (**V**) phosphate deposits in the region of injured myocardium (*n* = 6 per group; mean ± SD; ***P < 0.001, by 2-tailed, unpaired *t* test). Scale bars: 100 μm (**F** and **Q**) and 50 μm (**H** and **S**).

In *Abcc6*-KO injury, we observed calcific deposits to be initially formed in regions of cell death (Supplemental Figure 8). To determine whether *Abcc6* causes calcification in a myocyte-autonomous manner, we first deleted *Abcc6* conditionally (conditional knockout [CKO]) in cardiac muscle cells. For this purpose, we crossed *Myh6-Cre* mice with animals that had *Abcc6* alleles floxed to create *Myh6-Cre* *Abcc6*-CKO animals. We subjected the *Myh6-Cre Abcc6*-CKO animals to cardiac cryo-injury as described earlier (Figure 3A). However, we found that cardiac-specific deletion of *Abcc6* did not lead to cardiac calcification (Figure 3B). This finding was corroborated by CT imaging (Figure 3, C–E), Von-Kossa staining (Figure 3, F and G), immunofluorescence labeling of hydroxyapatite (Figure 3, H and I), and biochemical quantification of calcium and phosphate (Figure 3, J and K).

As the liver is known to express *Abcc6*, we next investigated whether liver-specific expression regulates cardiac calcification. For this purpose, we crossed animals expressing the liver-specific *albumin* *Cre* driver with *Abcc6^fl/fl^* animals to create progeny liver-specific *Abcc6*-CKO animals. The liver-specific *Abcc6*-CKO animals were maintained on a similar low-magnesium, high-phosphate diet and subjected to cryo-injury (Figure 3L). We observed extensive myocardial calcification in the liver-specific *Abcc6*-CKO animals (Figure 3M). Micro-CT imaging revealed calcified lesions in these animals (Figure 3, N–P). Histology with Von-Kossa staining and immunofluorescence staining confirmed the calcium deposits in injured cardiac tissue of liver-specific *Abcc6*-CKO animals (Figure 3, Q–T), and this was further corroborated by biochemical measurement of calcium and phosphate (Figure 3, U and V). These observations confirm that *Abcc6* acts in a hepatic cell–autonomous manner to affect ectopic cardiac calcification in *Abcc6*-deficient states and that tissue-specific deletion of *Abcc6* does not determine calcification in that tissue.

### Global Abcc6 deficiency leads to deficiencies in metabolites regulating nucleotide metabolism.

**Figure 4:**
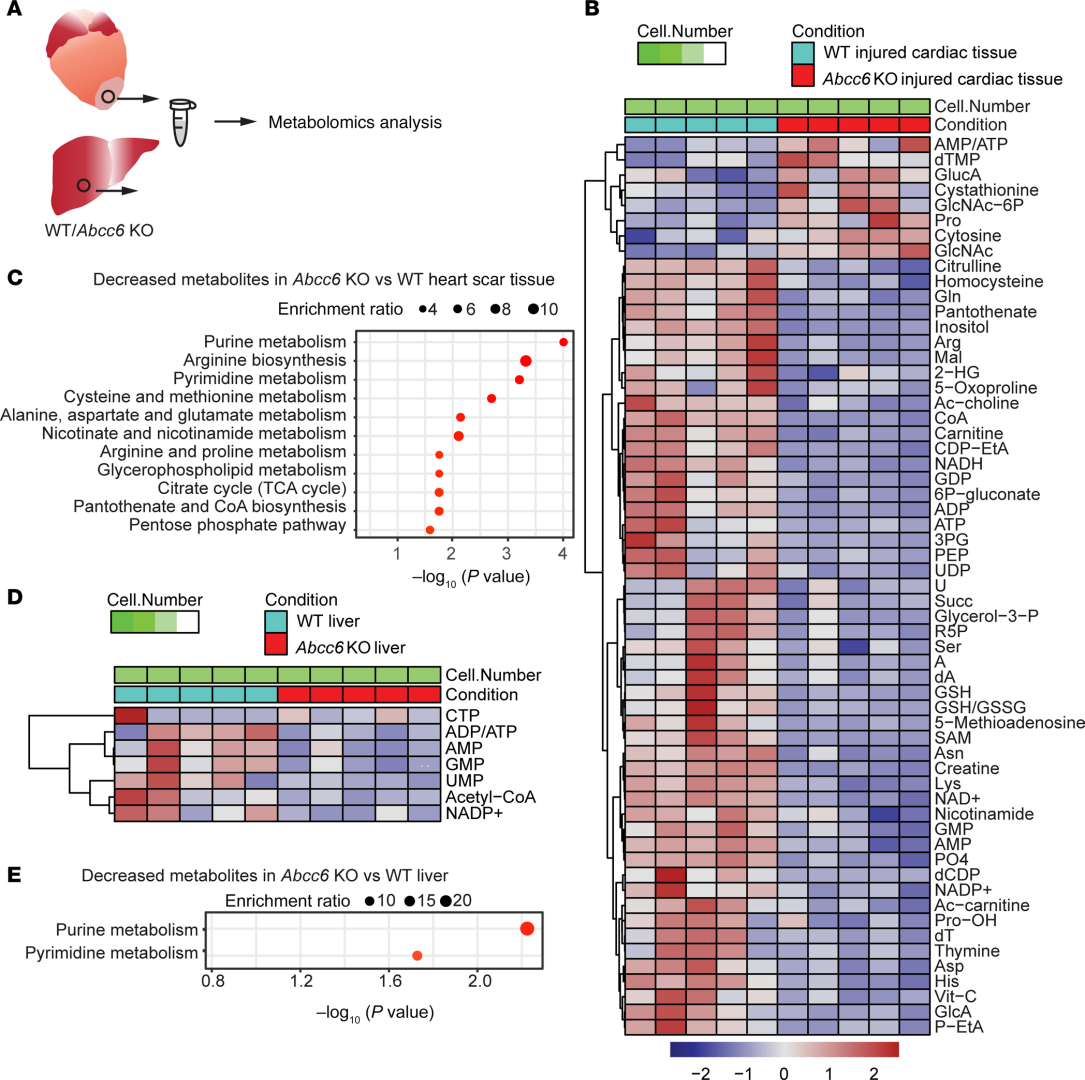
Loss of *Abcc6* alters metabolites in the injured myocardium. (**A**) Schematic of injured cardiac tissue and liver tissue collected from WT and *Abcc6*-KO animals for untargeted metabolomics 3 days following injury. (**B**) Untargeted metabolomics by liquid chromatography/mass spectrometry (LC/MS) of injured heart tissue from WT and *Abcc6*-KO animals at post–cryo-injury day 3, showing significantly altered metabolites in *Abcc6*-KO hearts versus WT hearts (*n* = 5 per group; *P < 0.05, **P < 0.01, ***P < 0.001, determined by one-way ANOVA. Significance is noted in Supporting Data Values). (**C**) KEGG analysis showing the top metabolic pathways significantly downregulated in injured *Abcc6*-KO hearts compared with WT. (**D**) Untargeted metabolomics by LC/MS of liver tissue from WT and *Abcc6*-KO animals at day 3 after cryo-injury of the heart, showing significantly altered metabolites (*P* < 0.05 (n=5 per group; *P < 0.05, **P < 0.01, ***P < 0.001 determined by one-way ANOVA) in *Abcc6*-KO livers versus WT livers. Significance is noted in the Supporting Data Values file. (**E**) KEGG analysis showing the top metabolic pathways significantly downregulated in *Abcc6*-KO liver tissue compared with WT after cardiac injury.

*Abcc6* is thought to be a metabolite transporter, although the exact identity of the metabolite(s) it transports remains unknown. To determine the metabolic abnormalities that could predispose to the development of cardiac calcification in *Abcc6*-deficient states, we first subjected animals with global *Abcc6* deficiency to cardiac cryo-injury and harvested the hearts of WT and *Abcc6*-KO animals on day 3 of cryo-injury for metabolomics analysis (Figure 4A). The predominant metabolic response was the depletion of metabolites affecting nucleotide metabolism (Figure 4B). Purine and pyrimidine nucleotides such as ATP, ADP, AMP, and GMP were downregulated in *Abcc6*-KO heart tissue (Figure 4B). Metabolites involved in cardiac energetics, particularly the NAD^+^ metabolite, were downregulated as well after cardiac injury. A Kyoto Encyclopedia of Genes and Genomes (KEGG) pathway analysis demonstrated metabolites related to purine, arginine, and pyrimidine metabolism to be significantly downregulated (Figure 4C). Taken together, these data indicate that aberrations in nucleotide metabolism were present in calcific tissues that underwent calcification in *Abcc6*-KO animals. As we have shown previously, the deficiency of the gene in the liver regulates cardiac calcification, we also harvested livers from *Abcc6*-KO mice (Figure 4A) and observed that similar metabolic abnormalities of nucleotide metabolism with decreased purine and pyrimidine metabolites were present in the liver as well (Figure 4, D and E).

### Liver-specific Abcc6 deficiency mirrors the metabolic abnormalities seen in animals globally deficient in Abcc6.

**Figure 5:**
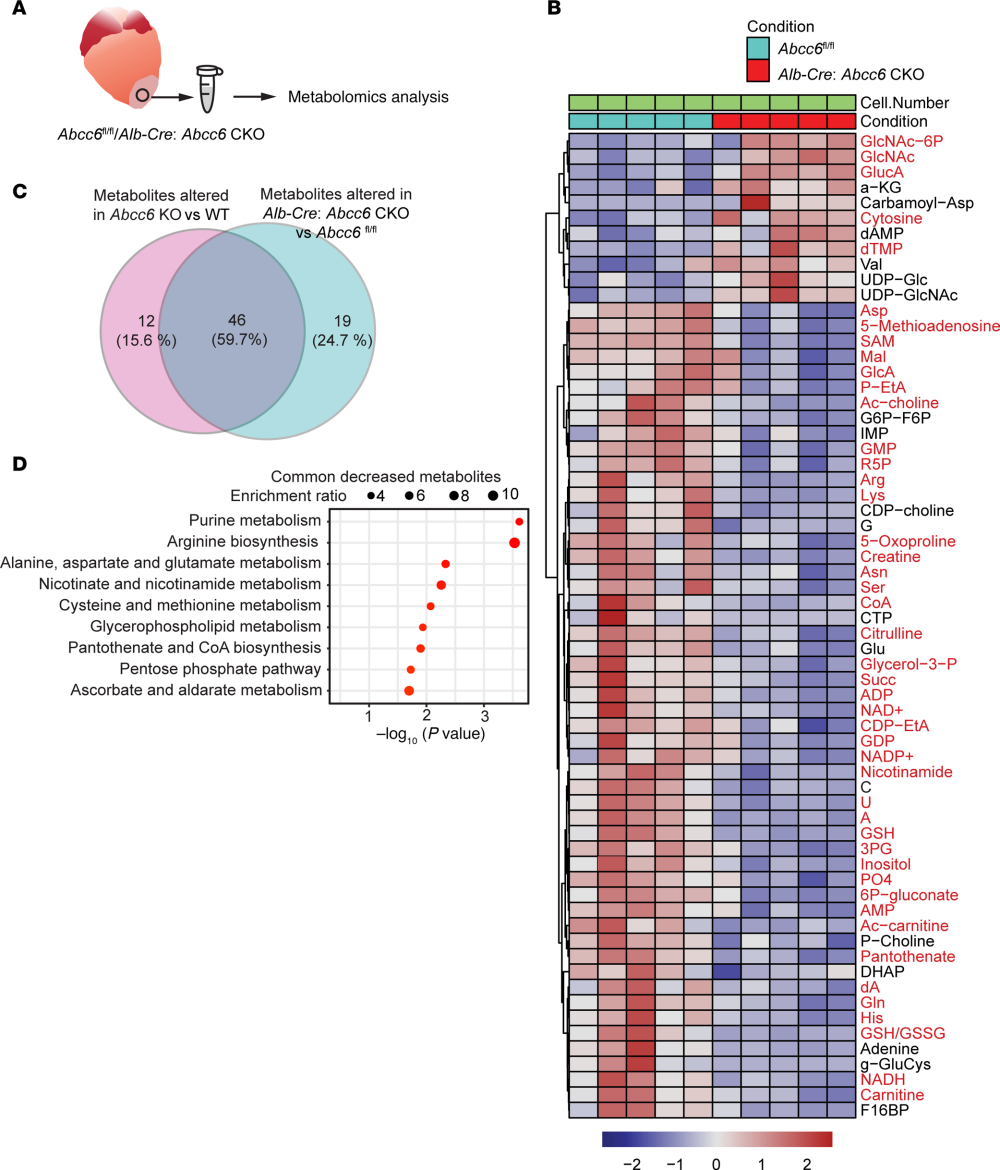
Liver-specific deletion of *Abcc6* leads to metabolic abnormalities in the injured heart similar to metabolic pathways affected in the injured hearts of *Abcc6*-KO animals. (**A**) Schematic of scar tissue collection from *Abcc6^fl/fl^* and *Alb-Cre*: *Abcc6*-CKO animals for metabolomic analysis. (**B**) Untargeted metabolomics by LC/MS of injured tissue from *Abcc6^fl/fl^* and *Alb-Cre* *Abcc6*-CKO animals at day 3 after cryo-injury, highlighting significantly altered metabolites in *Alb-Cre*: *Abcc6*-CKO hearts (*n*=5 per group; *P < 0.05, **P < 0.01, ***P < 0.001 determined by one-way ANOVA). Metabolites labeled in red overlap with those altered in injured hearts of global *Abcc6*-KO versus WT animals. (**C**) Venn diagram depicting metabolites differentially present between injured hearts of *Abcc6*-KO versus WT (left) and *Alb-Cre* *Abcc6*-CKO versus *Abcc6^fl/fl^* (right) mice. (**D**) KEGG analysis of metabolic pathways of the commonly altered metabolites that were significantly downregulated in injured tissue of *Abcc6*-KO versus WT and *Alb-Cre* *Abcc6*-CKO versus *Abcc6^fl/fl^* mice.

Our experiments demonstrate that liver-specific deletion of *Abcc6* led to ectopic cardiovascular calcification and recapitulated the phenotype observed in global *Abcc6*-KO animals. We next subjected liver-specific *Abcc6*-KO animals to cryo-injury and harvested the injured cardiac tissue for metabolomics analysis (Figure 5A), which demonstrated a significant reduction of nucleotides, including purines and pyrimidines. Purine, pyrimidine, and nicotinamide nucleotides were markedly downregulated in cardiac tissues of liver-specific *Abcc6*-CKO animals (Figure 5B). To identify the metabolites that were commonly dysregulated in injured heart tissue from global and liver-specific deletion in *Abcc6* animals, we compared the differentially up-/downregulated metabolites in each instance (Figure 5C). We observed that 60% of the metabolites were commonly dysregulated in injured heart tissues of animals that exhibited liver-specific *Abcc6* deletion and global *Abcc6* deletion (Figure 5C). KEGG pathway analysis demonstrated that nucleotide metabolism, including NAD and purine metabolism, was commonly downregulated in the hearts of liver-specific *Abcc6*-CKO and global-KO animals, demonstrating that liver-specific deletion of *Abcc6* affected similar metabolic pathways in the injured heart tissue as global *Abcc6* deficiency (Figure 5D). Taken together, these experiments suggest that the liver drives metabolic abnormalities in the injured heart tissue that create a permissive environment for ectopic calcification.

### The liver regulates cardiac mitochondrial function in an Abcc6-dependent manner.

**Figure 6:**
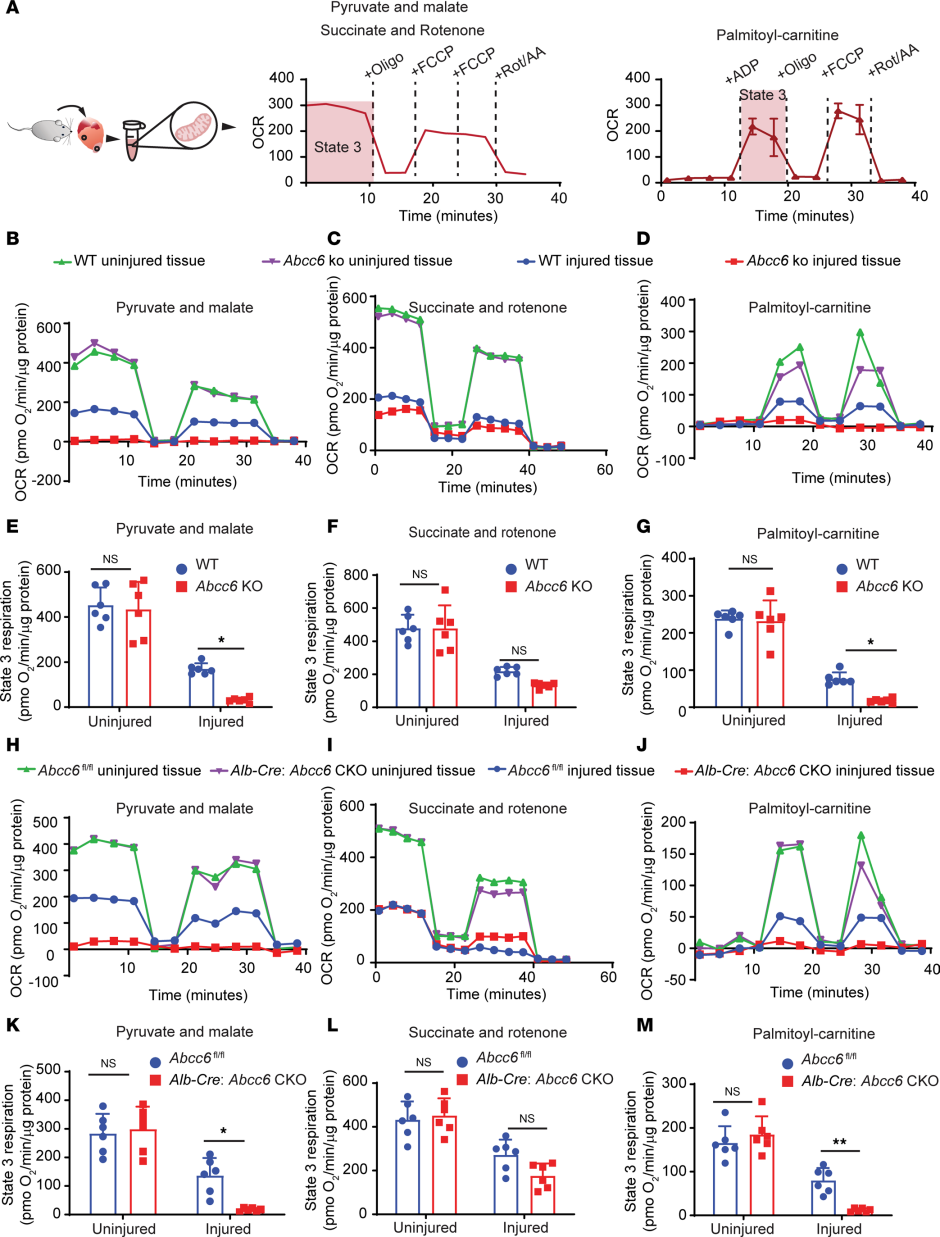
Mitochondrial oxygen consumption of injured heart tissue is reduced in global and liver-specific *Abcc6*-KO animals. (**A**) Schematic of Seahorse assays to measure mitochondrial oxygen consumption with the addition of complex-specific substrates and inhibitors. (**B**–**D**) Representative trace of OCRs in isolated mitochondria from uninjured and injured heart tissue of WT and *Abcc6*-KO animals treated with (**B**) complex I substrates (pyruvate and malate), (**C**) complex II substrate (succinate) and complex I inhibitor (rotenone), and (**D**) fatty acid oxidation substrates (palmitoyl-carnitine). In all cases, the OCR was normalized to the amount of mitochondrial protein loaded. (**E**–**G**) State 3 respiration quantification of isolated cardiac mitochondria from uninjured and injured cardiac tissue of WT and *Abcc6*-KO animals. Mitochondria were supplemented with (**E**) pyruvate and malate or (**F**) succinate and rotenone, or (**G**) palmitoyl-carnitine (*n* = 6 per group; mean ± SD; *P < 0.05, by 2-way ANOVA with Tukey’s multiple-comparison test). (**H**–**J**) Representative respirometry trace in isolated mitochondria from *Abcc6^fl/fl^* and *Alb-Cre*: *Abcc6*-CKO uninjured and injured cardiac tissue 3 days after surgery. Mitochondria were supplemented with (**H**) pyruvate and malate, (**I**) succinate and rotenone, or (**J**) palmitoyl-carnitine. (**K**–**M**) State 3 respiration of isolated cardiac mitochondria from uninjured and injured cardiac tissue of *Abcc6^fl/fl^* and *Alb-Cre* *Abcc6*-CKO animals. Mitochondria were treated with (**K**) substrates of pyruvate and malate, (**L**) succinate and rotenone, or (**M**) palmitoyl-carnitine (*n* = 6 per group; mean ± SD; *P < 0.05 and **P < 0.01, by 2-way ANOVA with Tukey’s multiple-comparison test).

Given the metabolic abnormalities noted in the injured heart in animals with global *Abcc6* deficiency, as well as in animals with liver-specific deletion of *Abcc6*, we next investigated the functional consequences of such metabolic abnormalities. Mitochondrial abnormalities have been noted in patients with PXE (26), and in animal models, calcification has been thought to originate around mitochondrial structures (17). To determine whether the mitochondria exhibited functional abnormalities in the injured heart in animals deficient in *Abcc6*, we subjected *Abcc6-*KO animals to cardiac cryo-injury and isolated mitochondria from their hearts at post-injury day 3 and measured oxygen consumption rates (OCRs) with the Seahorse analyzer. We focused on state 3 respiration, which in isolated mitochondria represents the mitochondria′s capacity to generate ATP when provided with specific substrates and ADP (27). We provided the isolated mitochondria substrates for complex I (pyruvate and malate), complex II (succinate with rotenone to inhibit complex I), and fatty acid oxidation (palmitoyl-carnitine) (Figure 6A). We observed that after adding the complex I electron transport chain substrates pyruvate and malate (28), there was no difference in OCRs between mitochondria obtained from uninjured hearts of WT and animals globally deficient in *Abcc6* (Figure 6B). However, mitochondria from injured hearts of *Abcc6*-KO animals exhibited a marked reduction in OCRs compared with mitochondria isolated from injured hearts of WT animals (Figure 6B). When complex II substrate was provided, no differences in OCRs were observed in the isolated mitochondria from injured heart tissue of WT and *Abcc6*-KO animals (Figure 6C). Similarly, mitochondria supplied with palmitoyl-carnitine, the substrate for fatty acid oxidation, showed decreased OCRs in isolated *Abcc6*-KO animals versus WT animals (Figure 6D). State 3 respiration was decreased in mitochondria from *Abcc6*-KO versus WT hearts when mitochondria were treated with pyruvate and malate or palmitoyl-carnitine but not succinate and rotenone (Figure 6, E–G). Taken together, the data suggest that differences in state 3 respiration were primarily due to abnormalities in complex I.

As liver-specific deletion of *Abcc6* was sufficient to induce cardiac calcification, we next examined whether liver-specific deletion of *Abcc6* leads to functional abnormalities of cardiac mitochondria. For this purpose, we isolated mitochondria from injured and uninjured hearts of animals with liver-specific *Abcc6*-CKO and respective Cre (–) *Abcc6^fl/fl^* controls. Measurement of the OCR demonstrated similar abnormalities in mitochondrial respiration rates, as noted in the animals with global *Abcc6* deficiency. There were no significant differences in OCRs in mitochondria isolated from uninjured hearts between the 2 groups, but mitochondria from injured hearts of liver-specific *Abcc6*-CKO animals showed a profound reduction in respiration with pyruvate and malate as the substrate (Figure 6H) but not with succinate and rotenone (Figure 6I).We observed a similar reduction in respiration with palmitoyl-carnitine (Figure 6J). Quantification of state 3 OCRs with the addition of complex I substrates confirmed the profound reduction of cellular respiration in mitochondria isolated from injured heart tissue from liver-specific *Abcc6*-CKO animals compared with Cre (–) controls (Figure 6K). State 3 OCRs with complex II substrate and complex I inhibitor did not show any significant abnormalities (Figure 6L). A similar reduction in the state 3 respiratory rate was also seen in mitochondria from injured hearts of animals with liver-specific *Abcc6* deletion, upon addition of palmitoyl-carnitine (Figure 6M). We also performed Western blotting and observed no change in the amount of oxidative phosphorylation proteins in isolated mitochondria from injured heart tissues of *Abcc6*-KO animals and their respective controls (Supplemental Figure 9). We did not observe impaired function of complex I in the mitochondrial electron transport chain, dependent on *Abcc6* in *Myh6-Cre* *Abcc6*-CKO animals, suggesting again that *Abcc6* acted in a nonmyocyte-autonomous manner (Supplemental Figure 10). These data, taken together, demonstrate that hepatic expression of *Abcc6*, likely through an altered systemic metabolic milieu, altered mitochondrial function in the heart following injury and suggest an altered systemic metabolic milieu as the basis of functional mitochondrial defects in PXE.

### The mineralization inhibitor clodronate prevents the development of cardiac calcification in animals deficient in Abcc6.

**Figure 7:**
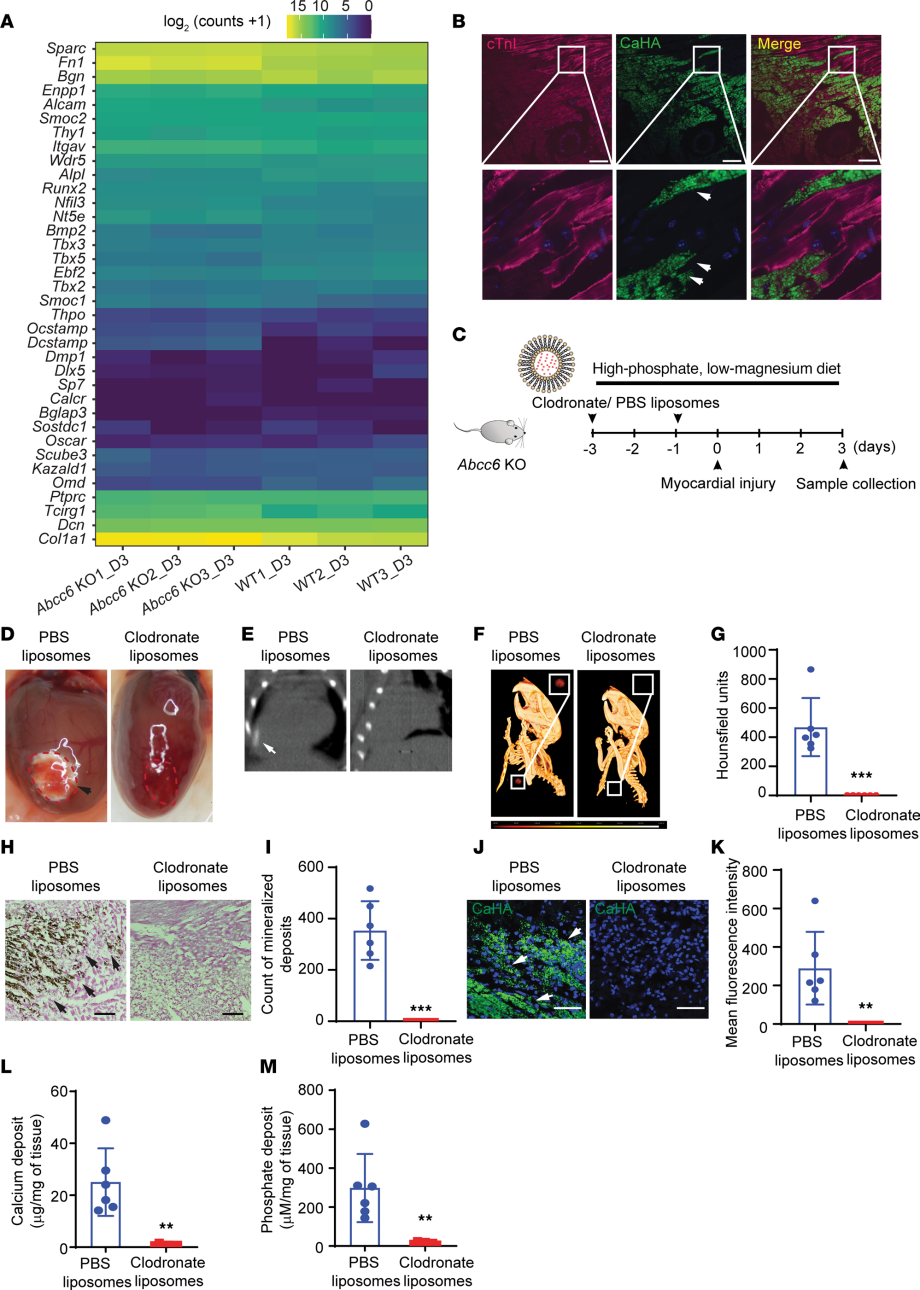
Clodronate inhibits cardiac calcification in animals deficient in *Abcc6*. (**A**) Heatmap showing the expression levels of a set of osteogenic genes in WT and *Abcc6*-KO injured heart tissue on day 3 after surgery demonstrating no significant differential expression (*n* = 3 in each group, *P* > 0.05). (**B**) Immunostaining for hydroxyapatite (green) and myocytes (red) in injured hearts from *Abcc6*-KO mice (inset shows high magnification). The arrow indicates the calcific area within the myocyte. Scale bars: 100 μm (inset: original magnification, ×60). (**C**) Experimental design for the administration of clodronate liposomes to *Abcc6*-KO animals to determine the effects on cardiac calcification. (**D**) Gross images 3 days after cryo-injury of hearts from *Abcc6*-KO mice that received PBS liposomes or clodronate liposomes (red dotted lines indicate the area of injury; arrowhead indicates the calcific lesion). Note that the animals that received clodronate liposomes showed no mineralization. (**E**) CT scan, (**F**) 3D rendering, and (**G**) quantitative analysis of calcium content in heart scar tissue (*n* = 6 in each group; mean ± SD; ***P < 0.001, by 2-tailed, unpaired Student’s *t* test comparing PBS liposome and clodronate liposome groups). (**H** and **I**) Histological staining of cryo-injured myocardium with (**H**) Von-Kossa staining and (**I**) corresponding quantitative analysis showing calcium deposits. Scale bars: 100 μm (*n* = 6 in each group; mean ± SD; ***P < 0.001, by 2-tailed, unpaired Student’s *t* test). (**J**) Immunostaining for hydroxyapatite and (**K**) corresponding quantitative analysis. Scale bars: 50 μm (*n* = 6 in each group; mean ± SD; **P < 0.01, by 2-tailed, unpaired Student’s *t* test). (**L** and **M**) Biochemical measurements of (**L**) myocardial calcium and (**M**) phosphate deposits in the injured region (*n* = 6 in each group; mean ± SD; **P < 0.01, by 2-tailed, unpaired Student’s *t* test, comparing PBS liposome and clodronate liposome groups).

Ectopic cardiac calcification is thought to parallel osteogenesis, with expression of the osteogenic transcriptional program in mesenchymal cells in the region undergoing ectopic calcification. In contrast, dystrophic calcification occurs after cell death or in scar tissue and represents mineralization of the matrix from hydroxyapatite mineral deposition and growth without an induction of an osteogenic transcriptional program. We first examined bulk sequencing gene expression data from injured heart tissue and examined expression levels of a panel of genes that are known to be associated with induction of an osteogenic transcription program, but we found no evidence of differential expression of osteogenic genes between animals with global *Abcc6* deficiency and WT controls (Figure 7A). Dystrophic calcification is initiated with cell death, and we performed immunostaining of the injured region and observed hydroxyapatite crystals within cardiomyocytes (Figure 7B). As dystrophic calcification is thought to be secondary to mineral crystallization and growth, we administered the first-generation bisphosphonate clodronate (1.8 mg/30 g of BW via i.p. injection) to determine the effects on cardiac calcification. *Abcc6*-KO animals were subjected to cardiac cryo-injury, and clodronate liposomes were administered 3 days and 1 day prior to cryo-injury (Figure 7C). On gross inspection, we observed complete rescue of calcification in the hearts of *Abcc6*-KO animals that had been administered clodronate liposomes compared with vehicle liposome–injected animals (Figure 7D). Micro-CT imaging of the heart and thorax showed an absence of calcium mineral deposits in the *Abcc6*-KO animals administered clodronate liposomes (Figure 7, E–G). Von-Kossa staining and immunofluorescence staining for hydroxyapatite confirmed the complete rescue of calcification in *Abcc6*-KO animals (Figure 7, H–K). Biochemical analysis demonstrated a significant reduction in calcium (Figure 7L) and phosphate (Figure 7M) deposition in injured hearts of animals injected with clodronate. Consistent with the known effects of liposomal clodronate on macrophage depletion (29), we observed significant depletion of both macrophages and neutrophils in the injured hearts of animals receiving clodronate liposomes versus vehicle (Supplemental Figure 11). To confirm the effects of bisphosphonate in reducing calcification in *Abcc6*-KO animals, we injected the bisphosphonate etidronate into *Abcc6*-KO animals subjected to cardiac cryo-injury. Etidronate is not known to deplete macrophages or neutrophils. Animals received 4 doses with each dose administered i.p. daily starting from the day prior to injury (Supplemental Figure 12A). On gross inspection, we observed complete rescue of cardiac calcification in animals that received etidronate (Supplemental Figure 12B), and CT scanning confirmed the absence of calcification in etidronate-injected animals (Supplemental Figure 12, C–E). Histological staining with Von-Kossa and immunofluorescence labeling of hydroxyapatite and biochemical measurements of calcium and phosphate confirmed the absence of calcification in injured hearts of animals that received etidronate (Supplemental Figure 12, F–K). Taken together, these data suggest that tissues undergo dystrophic calcification in *Abcc6*-deficient states and that mineralization inhibitors can attenuate or rescue the development of post-injury ectopic calcification.

## Discussion

Biallelic mutations in the gene *Abcc6* cause PXE, but the underlying biological mechanisms by which loss of function of *Abcc6* causes ectopic calcification of tissues such as the skin, eye, and cardiovascular system remain uncertain. The inhibitor hypothesis has been proposed to explain the mechanisms of calcification, in which loss of *Abcc6* results in decreased circulation of a putative inhibitor of mineralization. The balance of pro- and anti-mineralization factors is thus lost resulting in tissues undergoing ectopic calcification. In this regard, inorganic pyrophosphate levels have been noted to be abnormal in patients with PXE, and mutations in the gene ENPP1 that are associated with decreased PPi can lead to a PXE-like phenotype. However, there is no evidence that *Abcc6* is involved with PPi transport, and pyrophosphate levels in individuals with PXE do not correlate well with the phenotype. An analysis of *Abcc6* expression across all tissues demonstrated the highest expression of this gene in the liver, and although *Abcc6* is thought to be a transmembrane protein, recent evidence suggests that *Abcc6* could reside in mitochondria-associated endoplasmic reticulum membranes (MAMs) (30), which are specialized membranous structures linking mitochondria to the ER that play a role in lipid transport and calcium homeostasis. These findings have given rise to the notion that *Abcc6* may affect mitochondrial function, and individuals with PXE have abnormal mitochondrial structure and function, as shown in skin biopsy samples.

Our data suggest a central role of the liver in mediating calcification in *Abcc6*-deficient states. Deletion of *Abcc6* in the liver using the *albumin* Cre driver was sufficient to lead to ectopic calcification of the heart, whereas deletion of the gene in the cardiac muscle did not recapitulate the calcific phenotype. *Albumin* is predominantly expressed in the liver, but extrahepatic expression of *albumin* can be observed in certain conditions (31). Recent studies using overexpression of *Abcc6* in the liver with transfection approaches have also demonstrated an attenuation of the calcific phenotype in *Abcc6*-deficient animals, supporting a role of the liver in regulating the calcific phenotype in target organs (32). Taken together, these observations demonstrate that systemic effects of *Abcc6* were predominantly responsible for mediating ectopic calcification. Our data contrast with recent work, which demonstrated that loss of *Abcc6* in the liver was not sufficient to lead to whisker calcification (15). These discrepancies could arise from differences in the rapidity of the calcification process (72 hours for cardiac calcification vs. months for whisker calcification). Notwithstanding, our data clearly demonstrate that *Abcc6* acted in a target tissue nonautonomous manner to affect calcification. The injured calcific heart tissue in liver-specific *Abcc6*-KO animals had a large number of metabolites differentially present compared with the injured tissue of control animals, suggesting that the liver altered the systemic metabolic milieu that predisposed the tissue to calcification. Mitochondrial function is known to be abnormal in individuals with PXE, and our data suggest that the liver, probably through an altered metabolic milieu, affects the mitochondrial function of distant tissues. Although crosstalk between the heart and other organs has been previously described in heart failure (33), our data provide fresh evidence that hepatic *Abcc6* regulates mitochondrial function in the heart and potentially other organs that are affected by tissue calcification

Dystrophic calcification occurs secondarily to cell death rather than a cell-mediated process of ossification akin to osteogenesis (34–36). Mitochondrial calcium-handling defects have been known to be associated with abnormal calcium release. Mitochondrial functional defects in complex I of the electron transport chain were severe in animals that were both globally deficient in *Abcc6* or had liver-specific deficiency of *Abcc6*. Animal studies have demonstrated increased myocyte cell death after ischemic injury in *Abcc6*-KO animals (37). Metabolic pathways regulating purine, pyrimidine, arginine, and NAD pathways were affected in the injured hearts of animals with global or liver-specific *Abcc6* deficiency, and such an altered metabolic milieu could have led to functional defects in cardiac energetics with increased apoptosis, ROS, and abnormal calcium handling creating a permissive environment for calcification to occur. Our study has limitations, in that it did not precisely identify the metabolites/molecules secreted by the liver that inhibit ectopic calcification of the heart in an *Abcc6-*dependent manner. These molecules could represent classes of nucleotides or other lipids, secreted by the liver, that could affect target tissue mitochondrial function or affect the process of mineralization per se. Examining molecules that are differentially present or absent in the circulation of individuals with PXE and that are known to be secreted by the liver and affect cellular energetics or mineralization, could provide potential clues to the identity of metabolites that are secreted by the liver in a *Abcc6*-dependent manner to inhibit ectopic calcification. In this regard, nucleotide metabolites that were found differentially present in the injured hearts of *Abcc6*-KO and liver-specific *Abcc6*-CKO animals are known to affect ectopic calcification in humans. Metabolites such as AMP can be degraded by CD73 to form adenosine, which inhibits alkaline phosphatase critically regulating the final steps of ectopic calcium deposition. Thus, *Abcc6*, by regulating nucleotide metabolites in the injured tissue, could affect calcification in PXE. Clodronate and etidronate as bisphosphonates prevent mineralization growth and rescue the phenotype in *Abcc6*-deficient animals, and bisphosphonates have had some success in the treatment of ectopic calcification in PXE (38).

In summary, our study demonstrates that the liver, via *Abcc6* expression, regulates cardiac injury phenotypes in part by altering the systemic metabolic milieu and regulating cardiac mitochondrial function. These observations provide insight into mechanisms of liver-heart crosstalk in the orphan disease PXE.

## Methods

### Sex as a biological variable.

For mouse studies, both male and female animals were included, and comparable findings were observed in both sexes.

### Statistics.

All data are presented as the mean ± SD, and the value of *n* represents biological replicates. Statistical analysis was performed with GraphPad Prism 8.3 (GraphPad Software) using a 2-tailed, unpaired *t* test and 2-way ANOVA with Tukey multiple-comparison test as appropriate. A *P* value of less than 0.05 was considered statistically significant.

### Study approval.

All animal experiments followed protocols approved by UCLA’s IACUC. Animals were housed in the UCLA vivarium in compliance with the guidelines set by the American Association for Accreditation of Laboratory Animal Care.

### Data availability.

Detailed methods are provided in the Supplemental Methods. Values underlying graphed data in main and Supplemental figures are shown in the Supporting Data Values file. Uncropped blot images are provided as a supplemental file. Bulk RNA-seq data and sc-RNA-seq data were deposited in the Gene Expression Omnibus (GEO) database (GSE291855; https://www.ncbi.nlm.nih.gov/geo/query/acc.cgi?acc=GSE291855 and GSE312469; https://www.ncbi.nlm.nih.gov/geo/query/acc.cgi?acc=GSE312469). Additional requests may be sent to the corresponding author.

## Author contributions

YW performed experiments, collected, and analyzed the data, designed experiments, and contributed to writing of the manuscript. BS and BT performed animal surgeries. FM, YG, CP, and MP analyzed bulk RNA-seq and single-cell RNA-seq data and assisted with data interpretation and contextualization. ZZ, JK, TS, and AJL assisted with the animal irradiation. LZ and ZL assisted with TTC and sample collection. JTH and TG conducted the metabolomics studies. SX assisted with micro-CT studies. LS, LFDR, and OS performed the mitochondria respirometry and detection of OXPHOS expression. AD conceptualized the project, designed all experiments, supervised all data collection and analysis, and wrote the manuscript.

## Conflict of interest

The authors have declared that no conflict of interest exists.

## Funding support

This work is the result of NIH funding, in whole or in part, and is subject to the NIH Public Access Policy. Through acceptance of this federal funding, the NIH has been given a right to make the work publicly available in PubMed Central.

- NIH (AR075867, HL137241, HL149658, to AD).
- US Department of Defense (PR231375, to AD).
- American Heart Association (Postdoctoral Fellowship 906531, Career Development Award 24CDA1258673, to YW).
- National Center for Advancing Translational Sciences UCLA CTSI Grant (UL1TR001881, to AD and YW).

## Supplementary Material

Supplemental data

Unedited blot and gel images

Supporting data values

## Version 1 03/10/2026

In-Press Preview

## Version 2 05/01/2026

Electronic publication

## References

[1] Germain DP (2017). Pseudoxanthoma elasticum. Orphanet J Rare Dis.

[2] Ringpfeil F (2006). Pseudoxanthoma elasticum is a recessive disease characterized by compound heterozygosity. J Invest Dermatol.

[3] Bergen AA (2000). Mutations in ABCC6 cause pseudoxanthoma elasticum. Nat Genet.

[4] Le Saux O (2000). Mutations in a gene encoding an ABC transporter cause pseudoxanthoma elasticum. Nat Genet.

[5] Li Q (2014). Ectopic mineralization disorders of the extracellular matrix of connective tissue: molecular genetics and pathomechanisms of aberrant calcification. Matrix Biol.

[6] Mendelsohn G (1978). Cardiovascular manifestations of Pseudoxanthoma elasticum. Arch Pathol Lab Med.

[7] Nolte KB (2000). Sudden cardiac death owing to pseudoxanthoma elasticum: a case report. Hum Pathol.

[8] Combrinck M (2011). Pseudoxanthoma elasticum and sudden death. J Forensic Sci.

[9] Pfau K (2024). Pseudoxanthoma elasticum – Genetics, pathophysiology, and clinical presentation. Prog Retin Eye Res.

[10] Favre G (2017). The ABCC6 transporter: a new player in biomineralization. Int J Mol Sci.

[11] Korff S (2006). Fine mapping of Dyscalc1, the major genetic determinant of dystrophic cardiac calcification in mice. Physiol Genomics.

[12] Boraldi F (2013). Matrix gla protein and alkaline phosphatase are differently modulated in human dermal fibroblasts from PXE patients and controls. J Invest Dermatol.

[13] Van Gils M (2023). Inorganic pyrophosphate plasma levels are decreased in pseudoxanthoma elasticum patients and heterozygous carriers but do not correlate with the genotype or phenotype. J Clin Med.

[14] Ralph D (2023). Weighing the evidence for the roles of plasma versus local pyrophosphate in ectopic calcification disorders. J Bone Miner Res.

[15] Ziegler SG (2017). Ectopic calcification in pseudoxanthoma elasticum responds to inhibition of tissue-nonspecific alkaline phosphatase. Sci Transl Med.

[16] Ivandic BT (1996). A locus on chromosome 7 determines myocardial cell necrosis and calcification (dystrophic cardiac calcinosis) in mice. Proc Natl Acad Sci U S A.

[17] Korff S (2006). Calcification of myocardial necrosis is common in mice. Virchows Arch.

[18] Meng H (2007). Identification of Abcc6 as the major causal gene for dystrophic cardiac calcification in mice through integrative genomics. Proc Natl Acad Sci U S A.

[19] Drake MT (2008). Bisphosphonates: mechanism of action and role in clinical practice. Mayo Clin Proc.

[20] Abedin M (2004). Vascular calcification: mechanisms and clinical ramifications. Arterioscler Thromb Vasc Biol.

[21] Demer LL, Tintut Y (2014). Inflammatory, metabolic, and genetic mechanisms of vascular calcification. Arterioscler Thromb Vasc Biol.

[22] MacDonald KP (2010). An antibody against the colony-stimulating factor 1 receptor depletes the resident subset of monocytes and tissue- and tumor-associated macrophages but does not inhibit inflammation. Blood.

[23] Gordon SR (2017). PD-1 expression by tumour-associated macrophages inhibits phagocytosis and tumour immunity. Nature.

[24] Heidt T (2014). Differential contribution of monocytes to heart macrophages in steady-state and after myocardial infarction. Circ Res.

[25] Duffield JS (2005). Selective depletion of macrophages reveals distinct, opposing roles during liver injury and repair. J Clin Invest.

[26] Lofaro FD (2020). Relationship between mitochondrial structure and bioenergetics in *Pseudoxanthoma elasticum* dermal fibroblasts. Front Cell Dev Biol.

[27] Divakaruni AS, Jastroch M (2022). A practical guide for the analysis, standardization and interpretation of oxygen consumption measurements. Nat Metab.

[28] Karamanlidis G (2013). Mitochondrial complex I deficiency increases protein acetylation and accelerates heart failure. Cell Metab.

[29] Epelman S (2014). Embryonic and adult-derived resident cardiac macrophages are maintained through distinct mechanisms at steady state and during inflammation. Immunity.

[30] Martin LJ (2012). *ABCC*6 localizes to the mitochondria-associated membrane. Circ Res.

[31] Yoshida K (1997). Sequencing of cDNA encoding serum albumin and its extrahepatic synthesis in the Mongolian gerbil, Meriones unguiculatus. DNA Res.

[32] Brampton C (2014). The level of hepatic *ABCC*6 expression determines the severity of calcification after cardiac injury. Am J Pathol.

[33] Cao Y, t al (2022). Liver-heart cross-talk mediated by coagulation factor XI protects against heart failure. Science.

[34] Fournier DE (2020). Dystrophic calcification and heterotopic ossification in fibrocartilaginous tissues of the spine in diffuse idiopathic skeletal hyperostosis (DISH). Bone Res.

[35] Kim KM (1995). Apoptosis and calcification. Scanning Microsc.

[36] Zhao Y (2009). Characterization of dystrophic calcification induced in mice by cardiotoxin. Calcif Tissue Int.

[37] Mungrue IN (2011). *Abcc*6 deficiency causes increased infarct size and apoptosis in a mouse cardiac ischemia-reperfusion model. Arterioscler Thromb Vasc Biol.

[38] Moore SN (2015). Bisphosphonates: from softening water to treating PXE. Cell Cycle.

