# Supplementary material for: The liver regulates ectopic calcification in Abcc6-deficient models of pseudoxanthoma elasticum
Source: J Clin Invest. 2026 Mar 10;136(9):e193499. doi: 10.1172/JCI193499 (PMC13132379; doi:10.1172/JCI193499)
Supplement: Supplemental data [file jci-136-193499-s148.pdf]

The Liver Regulates Ectopic Calcification in *Abcc6*-deficient models of Pseudoxanthoma Elasticum

Yijie Wang[1,2,3,4,5,6], Baiming Sun[1,2,3,4,5,6], Feiyang Ma[3], Bo Tao[1,2,3,4,5,6], Yiqian Gu[3,7], Zhiqiang Zhou[1,8,9], Jason Kim[1], Linlin Zhang[1,2,3,4,5,6], Zhihao Liu[1,2,3,4,5,6], Johanna Ten Hoeve [6,10,11,12], Linsey Stiles [12,13,] Lucia Fernandez del Rio[13], Calvin Pan[1,8,9], Orian Shirihai [12,13], Shili Xu [11,12,14], Thomas Graeber[6 10,11] , Tamer Sallam[1,5], Matteo Pellegrini[3,4,14], Aldons J. Lusis[1,10,11], Arjun Deb*[1,2,3,4,5,6]

*Correspondence to: adeb@mednet.ucla.edu

The following are included in the supplementary material.

1) Supplemental Methods
2) References
3) Figures S1 to S12

Methods

Animal care and use

All animal experiments adhered to protocols approved by the Institutional Animal Care and Use Committee (IACUC) at UCLA. The animals were housed on a 12-hour light/dark cycle and had unrestricted access to food and water. Data were collected from male and female C57BL/6 strain animals aged 8 to 14 weeks.

Transgenic and mutant mice

Abcc6 KO mice were a gift from Dr. Bergen (1). *Myh6-Cre* mice (2) (Jackson Laboratory, # 011038) or *Alb-Cre* mice (3) (Jackson Laboratory, # 003574) were crossed with *Abcc6*$^{fl/fl}$ mice (4) (Jackson Laboratory, #030962) to generate *Myh6-Cre*: *Abcc6* CKO and *Alb-Cre*: *Abcc6* CKO progeny animals. CD45.1 mice were obtained from Jackson Laboratory (# 002014) (5). CD11b-DTR mice (6) (Jackson Laboratory, #006000) were crossed with *Abcc6* KO mice to generate CD11b-DTR: *Abcc6* KO progeny animals.

Myocardial Cryoinjury

Mice were placed on a phosphate-rich, magnesium-deficient diet(7) (Diet TD.00442; Harlan Teklad, Madison, WI) 3 days before surgery to promote calcification. The mouse model of myocardial cryoinjury was established as previously described (8). In brief, mice were anesthetized and intubated using a volume-cycled ventilator. A left thoracotomy was performed at the level of the second intercostal space. The exposed beating heart was subjected to cryoinjury by applying a 1 mm steel rod pre-cooled in dry ice for 10 seconds. Successful freezing was indicated by the immediate blanching of the tissue.

In Vivo Interventions

Abcc6 KO mice received anti-CSF1R monoclonal antibody (mAb; BioXcell, clone BE0213) to deplete macrophages(9, 10). Mice were administered 400 μg of antibody intraperitoneally 3 days before cryo-injury, followed by 100 μg 1 day before and 1 day after injury. An isotype-matched IgG control (BioXcell, BE0089) was administered on the same schedule. In CD11b-DTR: *Abcc6* KO mice, macrophages were depleted by intraperitoneal injection of diphtheria toxin (DT; 15 ng/g body weight) administered 3 days and 1 day before cryo-injury. PBS-injected mice served as controls. For the clodronate liposomes treatment experiment, WT and *Abcc6* KO animals were similarly subjected to cryo-injury. PBS or clodronate liposomes (Encapsula NanoSciences, CLD-8901) were given via intraperitoneal injection three days and one day prior to surgery at 1.8 mg / 30 g mouse (11). To investigate the effects of bisphosphonate on calcification, etidronate

disodium hydrate (Sigma, P5248) was administered intraperitoneally at 10 mg/kg/day. Treatment began 1 day prior to cryo-injury and continued daily until tissue harvest.

Calcification measurements

The mineral deposits in the cardiac scar area were quantified with colorimetric assays to measure calcium and phosphate levels, normalized to the weight of the excised tissues, as previously described (12). Briefly, the cardiac scar tissues were minced and decalcified overnight in 0.6 mM hydrochloric acid. 5 μl supernatant was mixed with 200 μl diluted working solution from Bioassay Systems QuantiChrom™ Calcium Assay Kit (Bioassay Systems DICA500) and incubated for 3 minutes. Optical density is read at 612 nm to assess the free calcium amount. For assessment of the phosphate amount, 200 μl supernatant or standard solution was mixed with 30 μl phosphate reagent from Phosphate Colorimetric Kit (Sigma, MAK030) and incubated for 30 minutes. Plot the standard curve based on the absorbance at 650 nm and determine the amount of phosphate.

In vivo microCT imaging

MicroCT imaging was performed as previously reported (13). Briefly, mice were anesthetized with 2% isoflurane and positioned in a temperature-controlled imaging chamber. Imaging was conducted using the CrumpCAT scanner (50 kVp, 200 μA,125 μm resolution, 720 projections, 80 ms/projection) (14). Images were reconstructed with a modified Feldkamp Algorithm. MicroCT datasets were analyzed using Amide (version 1.0.5) (15) and ORS Dragonfly (version 2022.2) (Dragonfly 2022.2. Comet Technologies Canada Inc., Montreal, Canada; https://dragonfly.comet.tech/). For quantification, a fixed-volume (6×6×4 mm) ROI and a Hounsfield Unit (HU) threshold of 350 (HU water = 0) were applied to the injured heart tissue. Volumetric calcium content (vHU) was calculated as the product of the mean HU value and the ROI volume (mm^3) (16).

Serum collection and chemistry analysis

Under anesthesia, whole blood was collected via retro-orbital puncture into tubes without anticoagulant. Samples were allowed to clot at room temperature for 30 minutes, followed by centrifugation at 2,000 × g for 10 minutes at 4°C to remove the clot. The resulting supernatant was collected as serum. Serum calcium, phosphorus, and magnesium concentrations were measured by IDEXX BioAnalytics.

Histological studies

Following being anesthetized with isoflurane, the animals were euthanized through cervical dislocation. Then the hearts were quickly dissected and then fixed in 4% paraformaldehyde in PBS at 4˚C overnight and series dehydrated by 10% and 30% sucrose. Afterwards, the hearts were embedded in OCT compound (Tissue-Tek).

Immunofluorescent staining was performed on 10 μm frozen sections. For immunofluorescence staining, tissue sections were post fixed in pre-chilled acetone at -20°C for 15 minutes, blocked in 10% species-specific serum in 1.5 % BSA/PBS for 1 hour, and incubated with primary antibodies diluted in 1% BSA/PBS at 4°C overnight. Secondary antibodies were diluted in PBS and incubated with the sections for 1 hour. Samples were counterstained with DAPI (1 μg/mL, Invitrogen, D3571) for 10 minutes and mounted with SlowFade Gold Antifade reagent (Invitrogen, S36936). To visualize the dead cells, the samples were stained with 2 μg/mL propidium iodide (Invitrogen, P3566) for 5 minutes. To visualize the hydroxyapatite, the samples were stained with 1:100 diluted OsteoImage^{TM} staining reagent (PA 1503, Lonza, Walkersville, MD, USA) before incubation with the primary antibody. Images were taken using Nikon Eclipse Ti2 confocal microscopy (Nikon, USA) and analyzed in NIS-Element AR software (Nikon) or ImageJ. For TTC staining to determine viable and dead myocardium in WT and *Abcc6* KO mice post injury, hearts were harvested 24 hours after cryo injury and cut into 1mm slices. Slices were incubated in 1% 2,3,5- Triphenyltetrazolium chloride (Sigma, T8877) solution for 30 minutes at 37°C in the dark.

Bone marrow transplantation

Eight-week-old recipient mice underwent lethal irradiation with 10 Gy from a cobalt source. For bone marrow harvesting, bone marrow was collected from male donor mice by flushing femurs and tibias with DMEM supplemented with 10% FBS and 5 U/mL heparin. Red blood cells were removed using RBC buffer (150 mmol/L NH_4Cl, 61 mmol/L $KHCO_3$, and 1 mmol/L Na_2EDTA, pH 7.3). The remaining cells were washed and resuspended in DMEM containing 1% bovine albumin. Each recipient mouse received an injection of 1×10^7 bone marrow cells in 0.1 mL via the tail vein (17).

Flow cytometry

Single-cell suspensions were generated from cardiac tissue as previously described (18), with minor modifications. Briefly, injured cardiac tissues were minced and digested in Tyrode's buffer (in mM: 136 NaCl, 5.4 KCl, 0.33 NaH_2PO_4, 1 $MgCl_2$, 10 HEPES, 0.18% glucose) containing 0.1 μg/mL liberase TH (Sigma, 5401151001). Samples were incubated at 37 °C for 30 minutes with gentle agitation. The digested tissue was passed through a 70-μm cell strainer and centrifuged at 500 × g for 3 minutes. The cell pellet was resuspended in 2 mL RBC lysis buffer and incubated for 10 minutes at room temperature, followed by immediate centrifugation at 500 × g for 5 minutes. Cells were washed and resuspended in PBS containing 1% FBS. For surface staining, cells were incubated with fluorophore-conjugated primary antibodies for 30 minutes at room temperature. Flow cytometry was performed using an Attune cytometer. Neutrophils were identified as

CD45$^+$CD11b$^+$Ly6G$^+$. Macrophages were identified as CD45$^+$CD11b$^+$Ly6G$^-$F4/80$^+$ (19). Data were analyzed using FlowJo v10.0.8.

For the determination of chimerism following transplantation, 100 µl of peripheral blood was collected in heparinized tubes to prevent clotting. Red blood cells were lysed in 2 mL of RBC lysis buffer for 10 minutes, followed by immediate centrifugation at 500 g for 5 minutes at room temperature. The remaining cells were stained with CD45.1 or CD45.2 antibodies in FACS buffer (1% BSA in PBS) for 30 minutes in the dark at room temperature. Before analysis, 5 µL of 7-AAD was added to the samples. Stained cells were assessed using a Canto II FACS instrument (BD Biosciences) with BD FACS DIVA software, and data were analyzed using FlowJo v.10.4 software.

Mitochondria isolation from the heart

For isolation of cardiac mitochondria, the heart was immediately removed and placed in ice-cold relaxation buffer (5 mM sodium pyrophosphate, 100 mM KCl, 5 mM EGTA, 5 mM HEPES; pH 7.4). The heart was squeezed with tweezers to remove blood, dissected to isolate the scar from the normal tissue, minced with scissors, and then placed in a 2 mL microcentrifuge tube with 0.5 mL of MSHE homogenization buffer (210 mM mannitol, 70 mM sucrose, 5 mM HEPES, 1 mM EGTA, pH 7.2). The heart tissue was homogenized using an ULTRA-TURRAX hand-held electric homogenizer. 1.5 mL of buffer was added to the homogenized tissue and centrifuged at 500 g for 8 minutes at 4 °C. The supernatant was removed, placed in a new tube, and centrifuged again at 700 g for 8 minutes at 4 °C. The supernatant was then transferred to 2 mL microcentrifuge tubes and centrifuged at 10,000 g for 10 minutes at 4 °C to pellet the mitochondria. The mitochondrial pellets were re-suspended in 20 µl ice-cold MSHE buffer and mitochondrial protein was measured with a Bicinchoninic Acid (BCA) protein assay kit (Pierce, 23227). The concentrated mitochondria were kept on ice to perform bioenergetics assays.

Protein gel electrophoresis and immunoblotting

5 µg of isolated mitochondria were mixed with the sample buffer containing β-mercaptoethanol. Samples were loaded into 4-12% Bis-Tris gels (ThermoFisher) and gel electrophoresis was performed in a mini-gel tank (ThermoFisher) with MES running buffer and under a constant voltage of 120 V. Proteins were transferred to a methanol-activated PVDF membrane in a Mini Trans-Blot cell (BioRad) at a constant voltage of 100V for 75 min on ice. Blots were blocked in 3% BSA in PBS-Tween20 (1 mL/L) for 1 h and incubated with the primary antibody overnight. Rodent OXPHOS cocktail antibody was used to study the mitochondrial ETC subunits, while TOMM20 was used as a mitochondrial mass marker. Membranes were then washed 3 times with PBS-T, incubated with the adequate HPR-conjugated secondary antibody, and washed 3 more times with PBS-T. Images were

acquired in a ChemiDoc Imaging System (BioRad) and band densitometry was quantified using Image Lab (BioRad).

Isolated Mitochondria Respirometry

All oxygen consumption measurements were conducted using an Agilent Seahorse XFe96 Analyzer. Oxygen consumption rates were measured in MAS buffer (70 mM sucrose, 220 mM mannitol, 5 mM KH_2PO_4, 5 mM $MgCl_2$, 1 mM EGTA, 2 mM HEPES, pH 7.2) supplemented with 0.1% BSA. Mitochondria were loaded into a XF96 microplate at 2 µg/well for palmitoyl-carnitine respiration (fatty acid oxidation), 1.5 µg/well for pyruvate and malate (complex I), and 1 µg/well for succinate and rotenone (complex II) in a volume of 20 µL per well. The plate was centrifuged at 2,100 g for 10 minutes at 4°C and then the volume was increased to 150 µL. For Complex I and Complex II driven respiration, the mitochondria were started in State 3 respiration with substrate and ADP, for fatty acid oxidation, ADP and palmitoyl-carnitine were injected in Port A. State 3 respiration was measured with 4 mM ADP in MAS buffer supplemented with 5 mM pyruvate with 1 mM malate, 40 µM palmitoyl-carnitine with 0.5 mM malate, or 5 mM succinate with 2 µM rotenone. Subsequent injections included oligomycin (final concentration of 3 µM), FCCP (4 µM), and rotenone and antimycin A (2 µM each). All oxygen consumption rates in isolated mitochondria were normalized to micrograms of total mitochondrial protein in the microplate well.

RNA extraction

Mice were anesthetized with isoflurane, followed by cervical dislocation post cryo injury day 3. The heart was rapidly excised, and the injured region was dissected and snap-frozen in liquid nitrogen. The tissue was homogenized in 1 mL of TRIzol (Invitrogen, 15596026) using ceramic beads with Bead Ruptor 24 homogenizer (Omni, 19-070). The aqueous phase was collected, mixed with ethanol, and processed using the Qiagen RNeasy Mini Kit (Qiagen, 74134) according to the manufacturer's instructions.

Bulk RNA sequencing and data analysis

Total RNA was used to generate RNA-seq libraries, which were sequenced on an Illumina HiSeq 3000 platform (single-end, 50 bp). The reads were mapped with STAR 2.7.11a (20) to the mouse genome (mm10) for the mouse cell libraries. The counts for each gene were obtained using quantMode GeneCounts in STAR commands, and the other parameters during alignment were set to default. Differential expression analyses were carried out using DESeq2 (21). Genes with adjusted P value < 0.05 were considered significantly differentially expressed. Significantly up-regulated or down-regulated genes were uploaded to the Enrichr (22) for the pathway analyses.

Single-cell RNA sample preparation, library preparation and sequencing

For preparation the single-cell RNA sequencing samples, 3 days post cryo injury WT and *ABCC6* KO mice scar regions of the heart were harvested and digested by 0.1 µg/mL liberase TH (Sigma, 5401151001). After digestion, cells were incubated with 10 µM calcein AM (Abcam, ab141420) and live cells were collected by flow cytometry. An equal number of cells from 3 mouse hearts were pooled in each group. For the generation of single-cell gel beads in emulsion, cells were loaded on a Chromium single cell instrument (10x Genomics) with an estimated targeted cell recovery of ~5,000 cells as per the manufacturer's protocol. In brief, single-cell suspension of cells in 0.4% BSA-PBS was added to each channel on the 10x chip. Cells were partitioned with Gel Beads into an emulsion in the Chromium instrument, where cell lysis and barcoded reverse transcription of RNA occurred following amplification.

Single-cell gene expression libraries were created using Chromium Next GEM Single Cell 3' (v3.1 Chemistry) (10x Genomics), Chromium Next GEM Chip G Single Cell Kit (10x Genomics), and Single Index Kit T Set A (10x Genomics) according to the manufacturer's instructions. Briefly, samples were loaded to target 10,000 cells to form GEMs and barcode individual cells. GEMs were then cleaned cDNA and libraries were also created according to the manufacturer's instructions. Library quality was assessed using the 4200 TapeStation System and D1000 ScreenTape (Agilent) and Qubit 2.0 (Invitrogen) for concentration and size distribution. Samples were sequenced using Novaseq 6000 (Illumina) using 100 cycles. 200 M reads were targeted for each sample, targeting 20,000 reads per cell. Raw reads were processed using the Cell Ranger Pipeline (10x Genomics).

Single-cell RNA-sequencing analysis

The Cellranger output expression matrices were merged for each sample. The R package Seurat (v4.3.0) was used to cluster the cells in the merged matrix. Cells with less than 100 genes or more than 10% of mitochondrial expression were first filtered out as low-quality cells. The NormalizeData function was used to normalize the expression level for each cell with default parameters. The FindVariableFeatures function was used to select variable genes with default parameters. The ScaleData function was used to scale and center the counts in the dataset. Principal component analysis (PCA) was performed on the variable genes. The RunHarmony function from the Harmony package was applied to remove potential batch effects among samples processed in different batches. Uniform Manifold Approximation and Projection (UMAP) dimensional reduction was performed using the RunUMAP function. The clusters were obtained using the FindNeighbors and FindClusters functions with the resolution set to 0.5. The cluster marker genes were found using the FindAllMarkers function. The cell types were annotated by overlapping the cluster markers with the published marker genes. The dot plot was plotted using the

DotPlot function. Differential expression analysis between two groups of cells was conducted using the FindMarkers function. Genes with adjusted p-value smaller than 0.05 were considered significantly differentially expressed.

LC-MS study

To analyze metabolites in the cardiac and liver tissue, 10–20 mg of tissue was collected for LC/MS. The samples were homogenized using a bead mill homogenizer (Bead Ruptor 24, Omni) in cold 80% MeOH at a ratio of 1 mL per 20 mg of tissue. The homogenized samples were then incubated at -80°C for 1 hour to enhance extraction efficiency and facilitate protein precipitation. Following incubation, the extracts were centrifuged at 16,000 g for 15 minutes at 4°C. The resulting supernatant was transferred to a glass vial and vacuum dried in preparation for LC-MS analysis.

LC-MS analysis

Vacuum-dried samples, prepared as described above, were resuspended in 50% ACN: water, and one-tenth of the sample was loaded onto a Luna 3 µm NH2 100Å (150 × 2.0 mm) column (Phenomenex). Chromatographic separation was carried out on a Vanquish Flex system (Thermo Scientific) using mobile phase A (5 mM NH_4AcO, pH 9.9) and mobile phase B (ACN) at a flow rate of 200 µL/min. A linear gradient from 15% A to 95% A over 18 minutes was followed by a 9-minute isocratic hold at 95% A and re-equilibration to 15% A.

Metabolite detection was performed using a Thermo Scientific Q Exactive mass spectrometer operating in polarity switching mode (+3.5 kV/−3.5 kV) with full-scan acquisition over an m/z range of 70–975 at a resolution of 70,000. Targeted metabolites were quantified using TraceFinder 4.1 (Thermo Scientific) based on area under the curve, with expected retention times and accurate mass measurements (<5 ppm). Relative metabolite levels were determined by summing the values of all isotopologues for each metabolite. Metabolite isotopologue distributions were corrected for natural ^{13}C abundance. Data analysis was conducted using in-house R scripts. Functional pathway enrichment of significantly altered metabolites was subsequently conducted using the Pathway Analysis module in MetaboAnalyst 6.0 (https://www.metaboanalyst.ca) based on the KEGG metabolic pathway database.

Antibodies

The following antibodies were used for immunostaining: goat anti-cardiac troponin I (1:100, Abcam, ab188877); rabbit anti-vimentin (1:100, Abcam, ab45939); Alexa Fluor 647 donkey anti-goat (1:200, Invitrogen, A21447); Alexa Fluor 594 donkey anti-rabbit (1:200, Invitrogen, A32754). The following antibodies were used for immunoblotting:

Rodent OXPHOS cocktail antibody (Abcam, ab110413), TOMM20 (Sigma, WH0009804M1). The following antibodies were used for FACS analysis: FITC anti-mouse CD45.1 Antibody (1:100, Biolegend, 110705); PE anti-mouse CD45.2 Antibody (1:100, Biolegend, 109807); Alexa Fluor 647 anti-mouse CD45 (1:400, Biolegend 103124), FITC anti-mouse CD11b (1:400, Biolegend 101206), Brilliant Violet 785™ anti-mouse Ly-6G (1:400, Biolegend 127645), PE anti-mouse F4/80 (1:400, Biolegend 123110).

Reference

1. Gorgels TG, Waarsing JH, Herfs M, Versteeg D, Schoensiegel F, Sato T, et al. Vitamin K supplementation increases vitamin K tissue levels but fails to counteract ectopic calcification in a mouse model for pseudoxanthoma elasticum. *J Mol Med (Berl).* 2011;89(11):1125-35.

2. Agah R, Frenkel PA, French BA, Michael LH, Overbeek PA, and Schneider MD. Gene recombination in postmitotic cells. Targeted expression of Cre recombinase provokes cardiac-restricted, site-specific rearrangement in adult ventricular muscle in vivo. *J Clin Invest.* 1997;100(1):169-79.

3. Postic C, Shiota M, Niswender KD, Jetton TL, Chen Y, Moates JM, et al. Dual roles for glucokinase in glucose homeostasis as determined by liver and pancreatic beta cell-specific gene knock-outs using Cre recombinase. *J Biol Chem.* 1999;274(1):305-15.

4. Ziegler SG, Ferreira CR, MacFarlane EG, Riddle RC, Tomlinson RE, Chew EY, et al. Ectopic calcification in pseudoxanthoma elasticum responds to inhibition of tissue-nonspecific alkaline phosphatase. *Sci Transl Med.* 2017;9(393).

5. Shen FW, Saga Y, Litman G, Freeman G, Tung JS, Cantor H, et al. Cloning of Ly-5 cDNA. *Proc Natl Acad Sci U S A.* 1985;82(21):7360-3.

6. Duffield JS, Forbes SJ, Constandinou CM, Clay S, Partolina M, Vuthoori S, et al. Selective depletion of macrophages reveals distinct, opposing roles during liver injury and repair. *J Clin Invest.* 2005;115(1):56-65.

7. Li Q, Kingman J, and Uitto J. Mineral content of the maternal diet influences ectopic mineralization in offspring of Abcc6(-/-) mice. *Cell Cycle.* 2015;14(19):3184-9.

8. Brunnert SR. Morphologic response of myocardium to freeze-thaw injury in mouse strains with dystrophic cardiac calcification. *Lab Anim Sci.* 1997;47(1):11-8.

9. MacDonald KP, Palmer JS, Cronau S, Seppanen E, Olver S, Raffelt NC, et al. An antibody against the colony-stimulating factor 1 receptor depletes the resident subset of monocytes and tissue- and tumor-associated macrophages but does not inhibit inflammation. *Blood.* 2010;116(19):3955-63.

10. Gordon SR, Maute RL, Dulken BW, Hutter G, George BM, McCracken MN, et al. PD-1 expression by tumour-associated macrophages inhibits phagocytosis and tumour immunity. *Nature.* 2017;545(7655):495-9.

11. Hulsmans M, Clauss S, Xiao L, Aguirre AD, King KR, Hanley A, et al. Macrophages Facilitate Electrical Conduction in the Heart. *Cell.* 2017;169(3):510-22 e20.

12. Pillai ICL, Li S, Romay M, Lam L, Lu Y, Huang J, et al. Cardiac Fibroblasts Adopt Osteogenic Fates and Can Be Targeted to Attenuate Pathological Heart Calcification. *Cell Stem Cell.* 2017;20(2):218-32 e5.

13. Tamboline M, Collins J, Jackson W, Gu W, Worssam M, Cheng P, et al. Preclinical evaluation of high-resolution CT, (18)F-FDG, and (18)F-NaF PET imaging for longitudinal monitoring of atherosclerosis. *Eur J Nucl Med Mol Imaging.* 2025;52(11):4256-67.

14. Taschereau R, Chatziioannou AF, and Xu S. Retrospective Cardiac Gating with A Prototype Small-Animal X-ray Computed Tomograph. *J Vis Exp.* 2025(216).

15. Loening AM, and Gambhir SS. AMIDE: a free software tool for multimodality medical image analysis. *Mol Imaging.* 2003;2(3):131-7.

16. Hsu JJ, Lu J, Umar S, Lee JT, Kulkarni RP, Ding Y, et al. Effects of teriparatide on morphology of aortic calcification in aged hyperlipidemic mice. *Am J Physiol Heart Circ Physiol.* 2018;314(6):H1203-H13.

17. Shi W, Wang X, Tangchitpiyanond K, Wong J, Shi Y, and Lusis AJ. Atherosclerosis in C3H/HeJ mice reconstituted with apolipoprotein E-null bone marrow. *Arterioscler Thromb Vasc Biol.* 2002;22(4):650-5.

18. Wang Y, Li Q, Tao B, Angelini M, Ramadoss S, Sun B, et al. Fibroblasts in heart scar tissue directly regulate cardiac excitability and arrhythmogenesis. *Science.* 2023;381(6665):1480-7.

19. Simoes FC, Cahill TJ, Kenyon A, Gavriouchkina D, Vieira JM, Sun X, et al. Macrophages directly contribute collagen to scar formation during zebrafish heart regeneration and mouse heart repair. *Nat Commun.* 2020;11(1):600.

20. Dobin A, Davis CA, Schlesinger F, Drenkow J, Zaleski C, Jha S, et al. STAR: ultrafast universal RNA-seq aligner. *Bioinformatics.* 2013;29(1):15-21.

21. Love MI, Huber W, and Anders S. Moderated estimation of fold change and dispersion for RNA-seq data with DESeq2. *Genome Biol.* 2014;15(12):550.

22. Kuleshov MV, Jones MR, Rouillard AD, Fernandez NF, Duan Q, Wang Z, et al. Enrichr: a comprehensive gene set enrichment analysis web server 2016 update. *Nucleic Acids Res.* 2016;44(W1):W90-7.

Figure S1

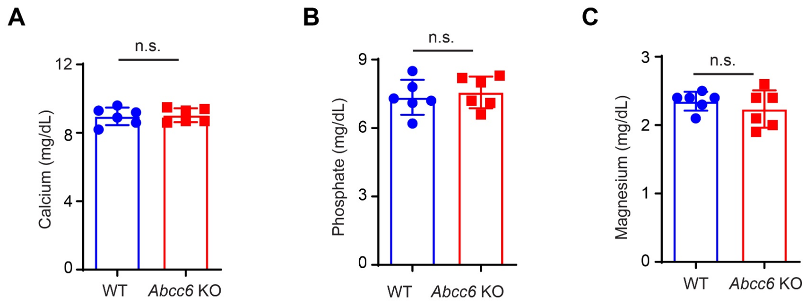

Figure S1. Serum calcium, phosphorus and magnesium levels in WT versus *Abcc6* KO animals. Measurement of serum (**A**) calcium, (**B**) phosphate and (**C**) magnesium. (n = 6 per group; mean± SD.; P value was calculated by two-tailed unpaired t-test, n.s.: not significant).

Figure. S2

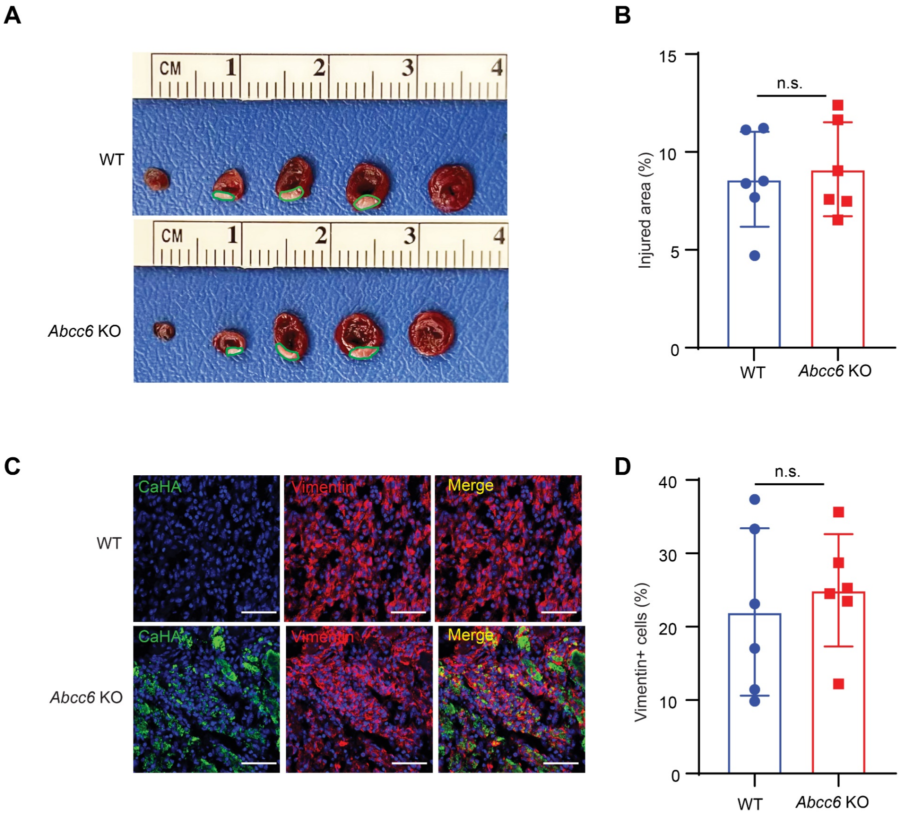

Figure S2. Cell death in WT and *Abcc6* KO hearts after cardiac cryo-injury. (**A**) TTC staining showing viable (red) and dead myocardium (outlined with a green line) in WT and *Abcc6* KO hearts 24 hours after cryo-injury (sections taken from base to apex of the injured area). (**B**) Quantification of injured area from TTC staining (n = 6 per group). (**C**) Immunostaining for fibroblasts (identified by vimentin) in WT and *Abcc6* KO hearts 3 days post-injury, and (**D**) quantification of fibroblasts. Scale bar: 50 µm. (n = 6 per group; mean ± SD.; n.s., not significant; WT versus *Abcc6* KO; *P* values calculated by two-tailed unpaired t-test).

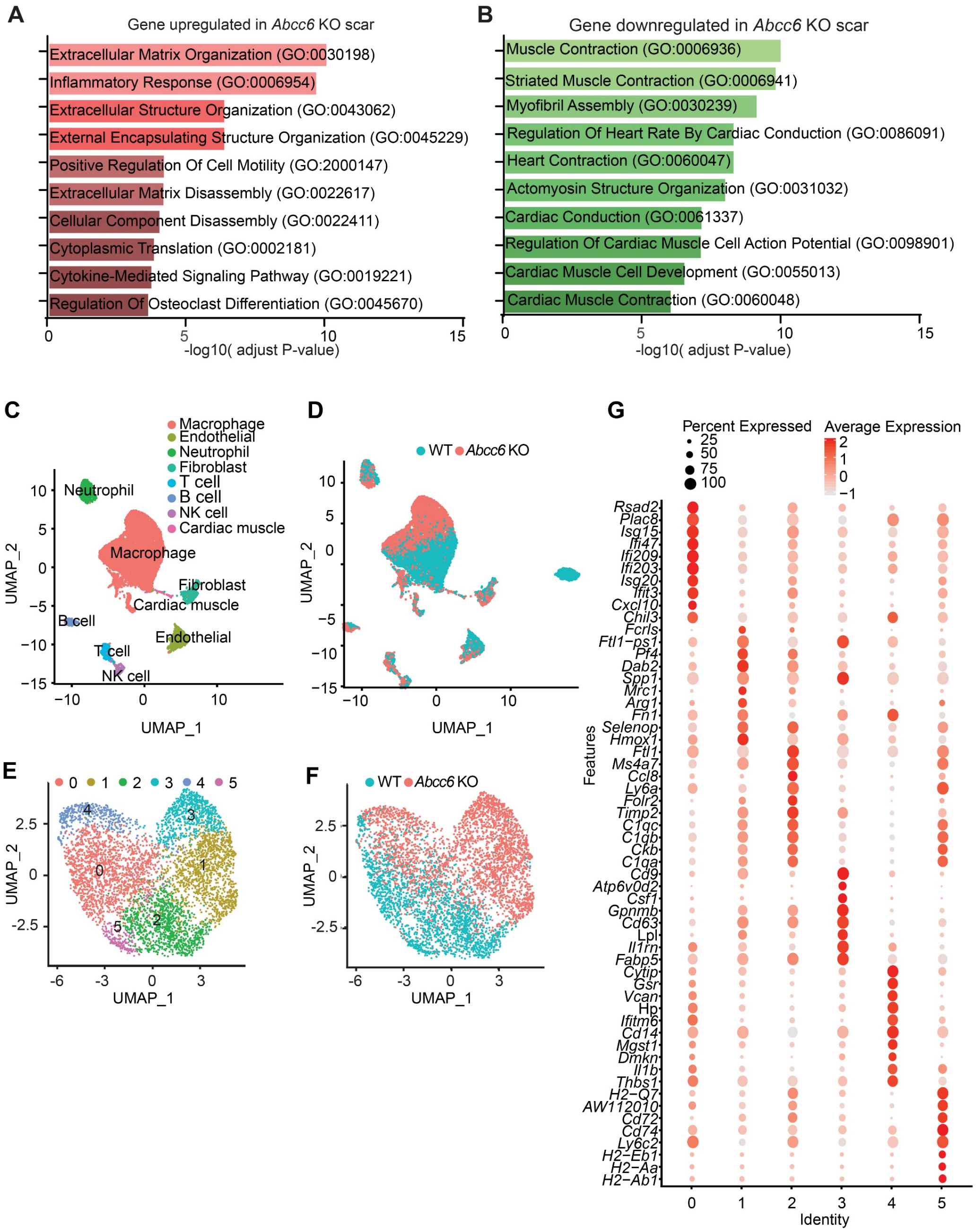

Figure S3. Bulk RNA and single-cell RNA sequencing of injured cardiac tissue in WT and *Abcc6* KO animals 3 days post–cryo-injury. (**A–B**) Gene ontology analysis of pathways differentially (**A**) upregulated or (**B**) downregulated in *Abcc6* KO versus WT hearts. (**C**) UMAP plot showing major cell populations in the cryo-injured heart and (**D**) proportion of each cell type in WT and *Abcc6* KO hearts (n = 3 per group). (**E**) UMAP

plot showing sub-clustering of macrophages and (**F**) distribution of macrophage subclusters across WT and *Abcc6* KO genotypes. (**G**) Dot plot showing marker genes used to identify macrophage subclusters.

Figure S4

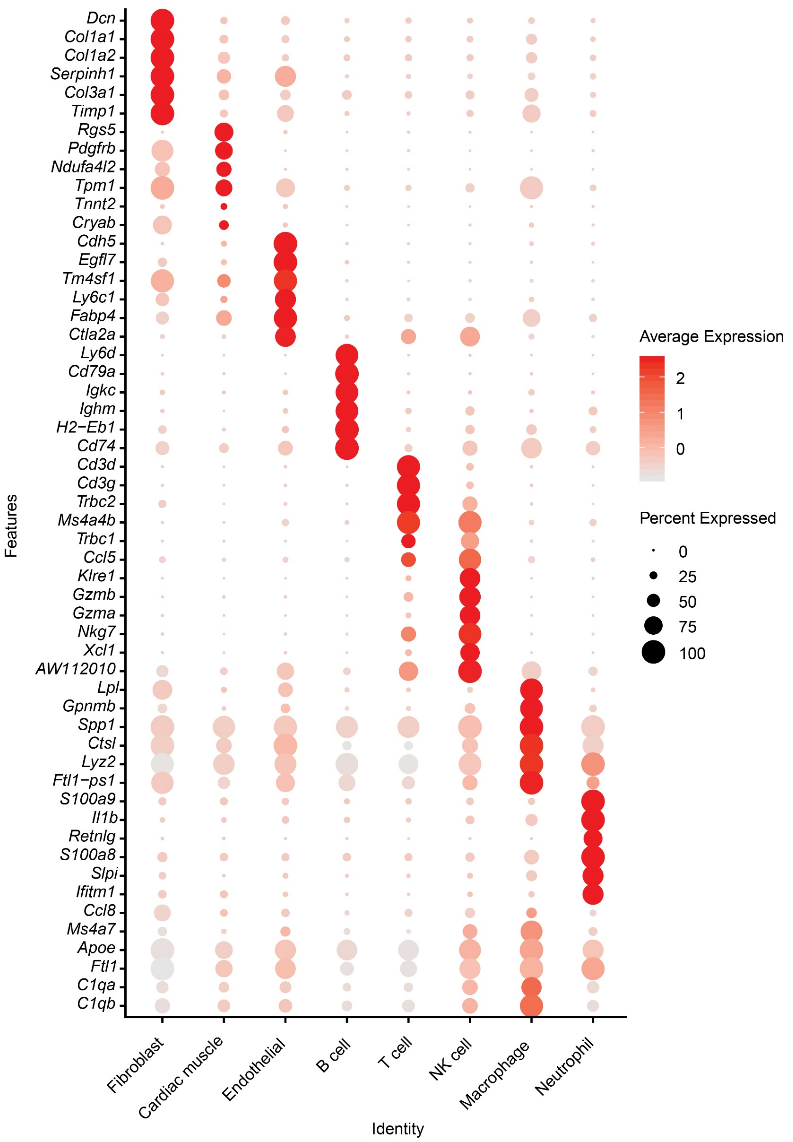

Figure S4. Dot plot representing the expression of marker genes that are expressed in specific cell types. Cell populations in the injured heart were identified according to the expression of canonical genes expressed in the different cell populations.

Figure S5

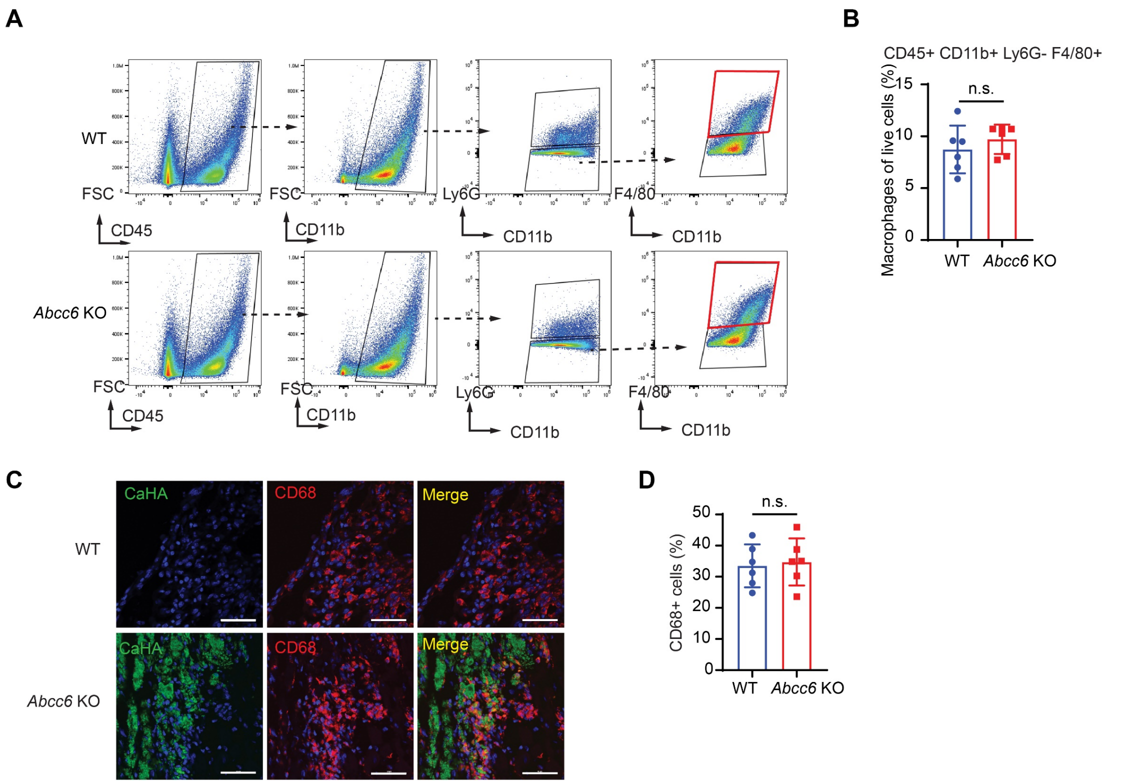

Figure S5. Macrophage infiltration in injured cardiac tissue of WT and *Abcc6* KO animals.

(**A**) Representative flow cytometry plots and gating strategy for macrophages in cryo-injured WT and *Abcc6* KO mouse hearts (red box indicates the macrophage population). (**B**) Quantification of macrophages showing similar infiltration between WT and *Abcc6* KO animals. (**C**) Immunofluorescent staining of CD68 in mouse hearts harvested 3 days post cryo injury and (**D**) quantification of $CD68^+$ cells demonstrating comparable macrophage presence in scar tissue. Scale bar: 50 μm. (n = 6 per group; mean ± SD.; n.s., not significant; WT versus *Abcc6* KO groups; *P* values calculated by two-tailed unpaired t-test).

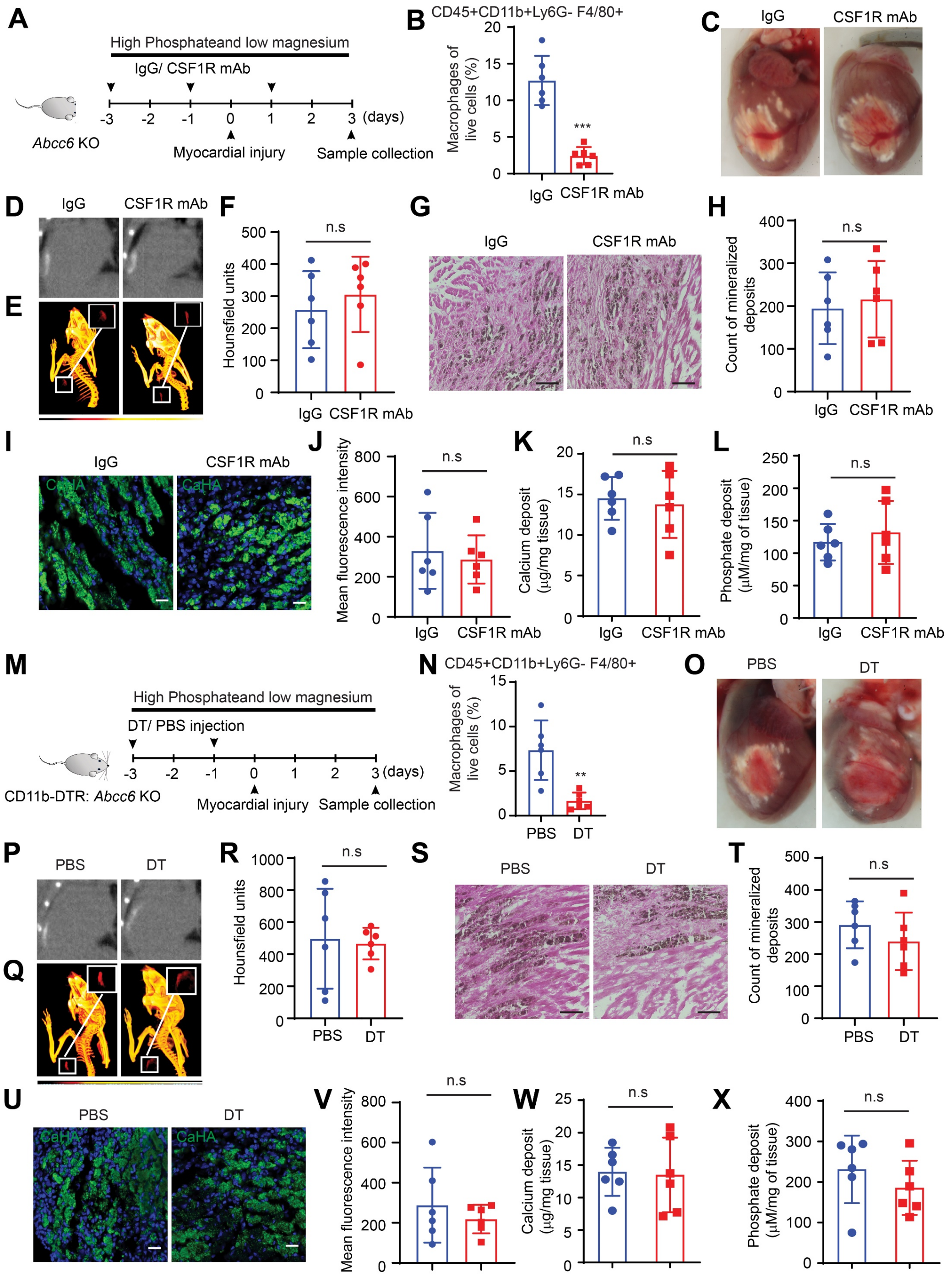

Figure S6 Macrophage depletion in *Abcc6* KO animals does not alter cardiac calcification following cryo-injury.

(**A**) Schematic of the experimental strategy showing administration of anti-CSF1R monoclonal antibody in *Abcc6* KO animals to assess effects on cardiac calcification. (**B**) Quantification of macrophages showing reduced infiltration in *Abcc6* KO animals

receiving anti-CSF1R antibody (n = 6 per group; mean ± SD.; n.s., IgG versus anti-CSF1R; *P* values by two-tailed Student's t-test). (**C**) Gross images of hearts 3 days post–cryo-injury from *Abcc6* KO animals treated with IgG or anti-CSF1R antibody. (**D**) CT scan, (**E**) 3D rendering and (**F**) quantification of calcium content in scar tissue based on CT scan data. (**G–H**) Histological analysis with (**G**) Von Kossa staining and (**H**) corresponding quantification showing calcium deposits (scale bar: 100 μm). (**I–J**) Immunostaining for (**I**) hydroxyapatite and (**J**) quantification (scale bar: 50 μm). (**K–L**) Biochemical measurements of myocardial (**K**) calcium and (**L**) phosphate in injured regions (n = 6 per group; mean ± SD.; n.s., IgG versus anti-CSF1R antibody; *P* values by two-tailed Student's t-test). (**M**) Experimental design of cryo-injury in CD11b-DTR: *Abcc6* KO animals receiving PBS or diphtheria toxin (DT). (**N**) Quantification of macrophages showing reduced infiltration in CD11b-DTR: *Abcc6* KO animals treated with PBS or DT (n = 6 per group; mean ± SD.; n.s., PBS versus DT; *P* values by two-tailed Student's t-test). (**O**) Gross images of cryo-injured hearts 3 days post-injury. (**P**) CT scan, (**Q**) 3D rendering and (**R**) quantification of calcium content in scar tissue. (**S–T**) Histological analysis with (**S**) Von Kossa staining and (**T**) quantification showing calcium deposits (scale bar: 100 μm). (**U–V**) Immunostaining for (**U**) hydroxyapatite and (**V**) quantification (scale bar: 50 μm). (**W–X**) Biochemical measurements of myocardial (**W**) calcium and (**X**) phosphate in injured regions (n = 6 per group; mean ± SD.; n.s., two-tailed unpaired t-test).

Figure S7

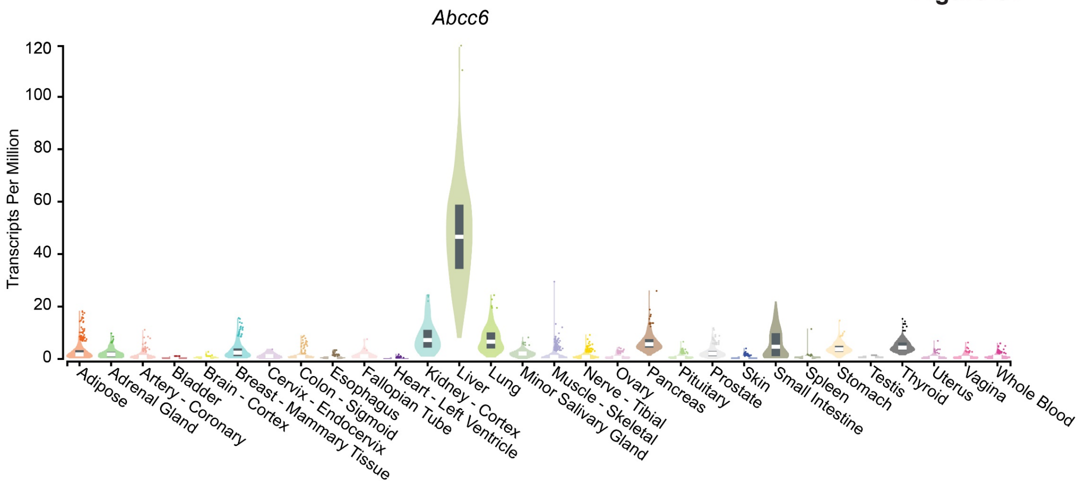

Figure S7. Comparison of *ABCC6* gene expression levels across 30 human tissues in the Genotype tissue expression (GTEx) program. Notably, the liver exhibits the highest *Abcc6* expression among all tissues.

Figure S8

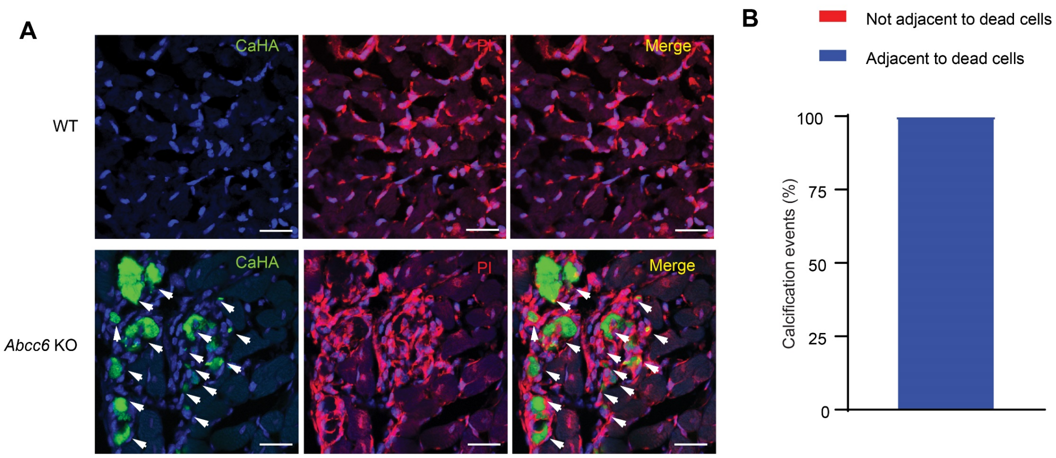

Figure S8. Cell death occurs in calcified areas of *Abcc6* KO animals after cardiac cryo-injury. (A) Immunostaining showing that calcified regions co-localize with areas of cell death, identified by propidium iodide (PI) staining. **(B)** Quantification demonstrating that calcification occurred exclusively within PI^+ (dead) areas and was absent in viable tissue. Scale bar: 25 µm. (n = 6 per group, Data are presented as mean ± SD.)

Figure S9

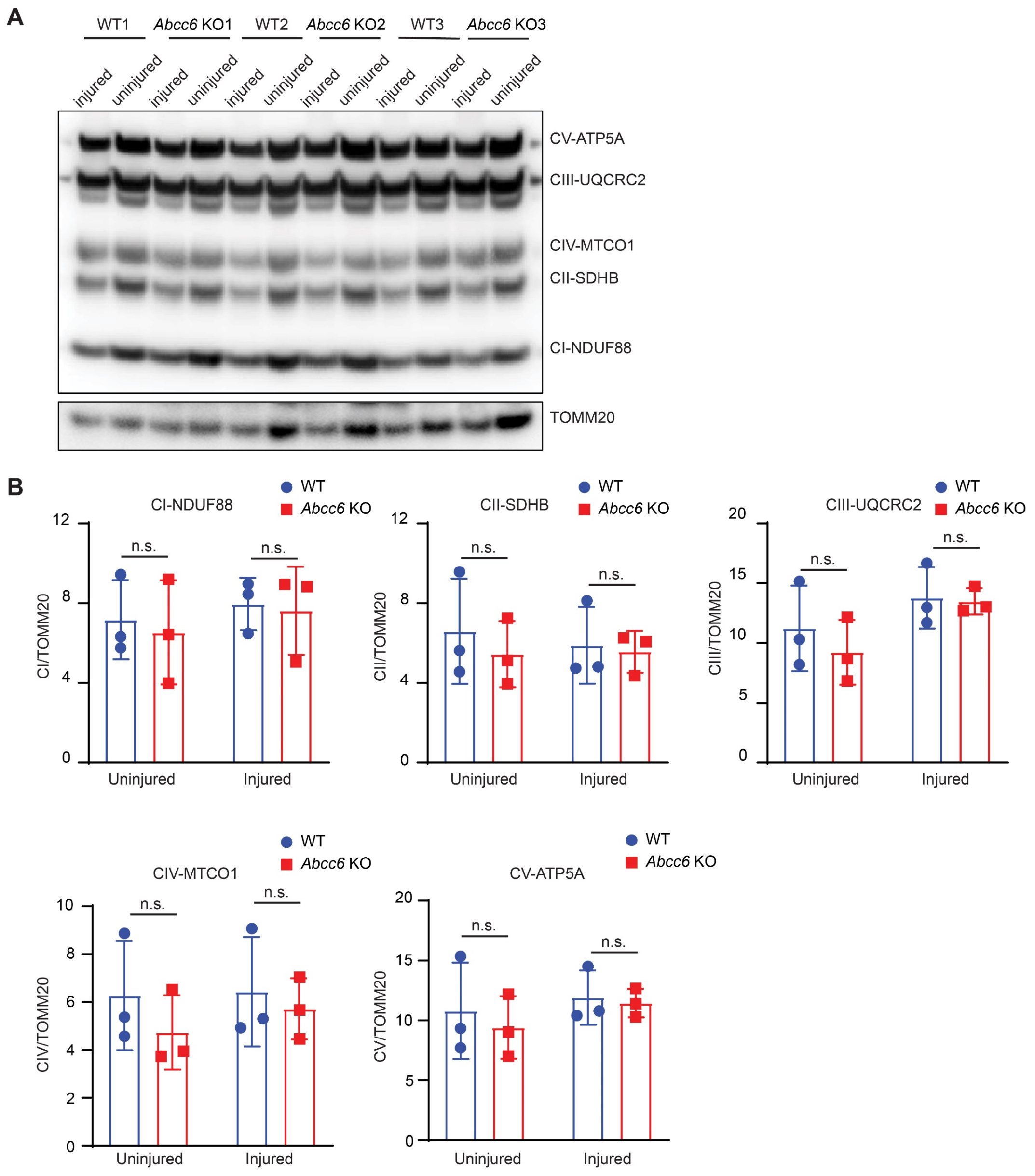

Figure S9. Oxidative phosphorylation protein levels are not affected in mitochondria isolated from injured heart tissue of *Abcc6* KO animals.

(**A**) Western blot analysis and (B) quantitative densitometry showing OXPHOS protein levels in the scar and normal regions of the heart in WT and *Abcc6* KO mice at 3 days post-cryo injury. (3 animals in each group. (CI-V) Refer to the electron transport chain complexes. Data are expressed as mean± SD.; n.s.: not significant. Statistics were determined using two-way ANOVA with Tukey's multiple comparisons test.

Figure S10

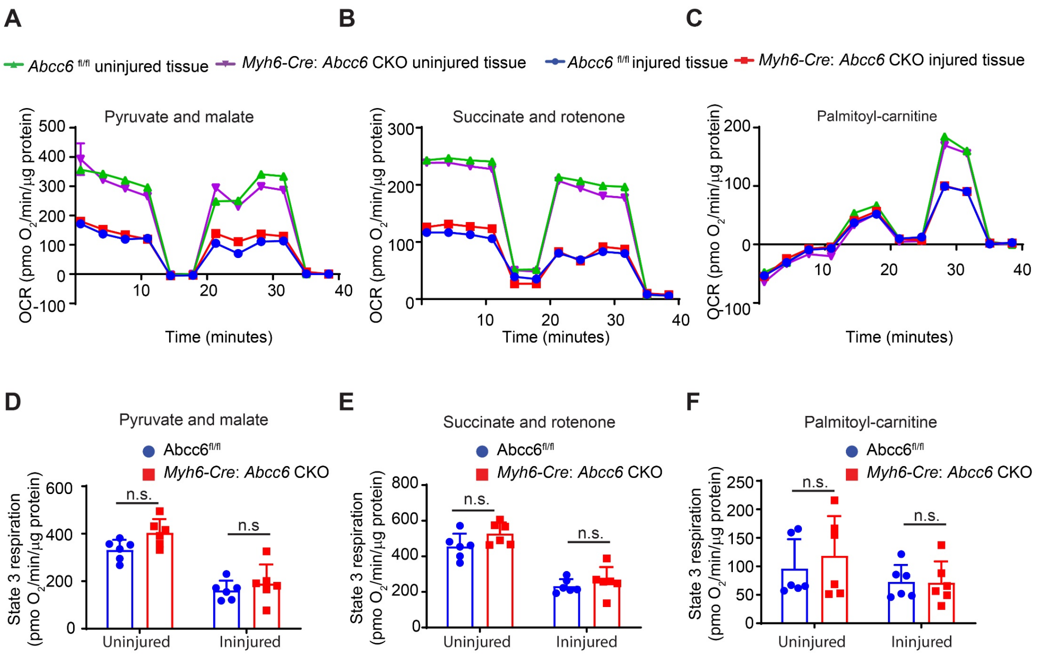

Figure S10. Oxygen consumption rates of mitochondria isolated from injured hearts of cardiac-specific *Abcc6* CKO animals and *Abcc6*$^{fl/fl}$ controls. (**A-C**) Representative Seahorse traces showing oxygen consumption rates (OCR) from mitochondria isolated from injured and uninjured heart tissue of *Abcc6*$^{fl/fl}$ and *Myh6-Cre*: *Abcc6* CKO animals. Assays started with (**A**) complex I substrates (pyruvate and malate); (**B**) complex II substrate (succinate) and complex I inhibitor (rotenone); or (**C**) fatty acid oxidation substrate (palmitoyl-carnitine). In all cases, OCR is normalized to mitochondrial protein loaded per well. (**D-F**) Quantification of state 3 respiration from the previously depicted Seahorse experiment measuring mitochondria isolated from injured and uninjured hearts of *Abcc6*$^{fl/fl}$ and *Myh6-Cre*: *Abcc6* CKO animals uninjured and injured cardiac tissue. Assays were started with (**D**) pyruvate and malate; (**E**) succinate and rotenone; or (**F**) palmitoyl-carnitine (*n*= 6 per group). Data are expressed as mean±SD., n.s.: not significant. Statistics were determined using two-way ANOVA with Tukey's multiple comparisons test.

Figure S11

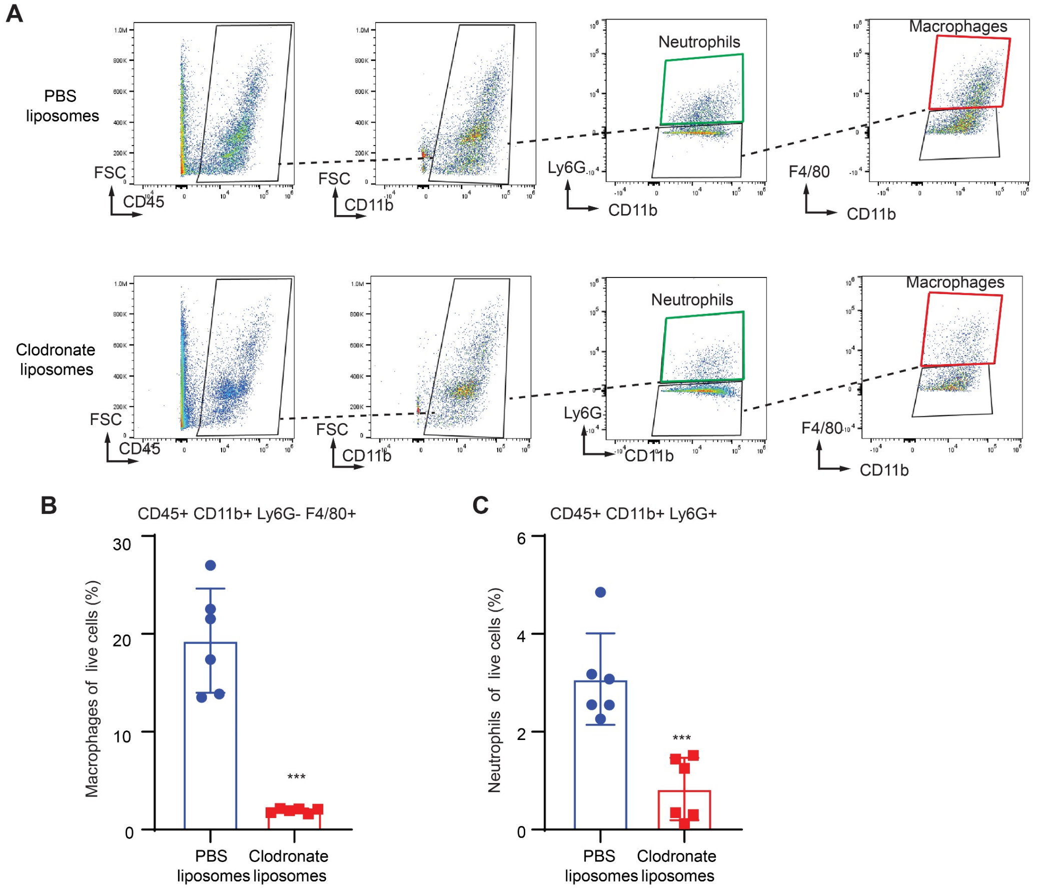

Figure S11. Macrophage and neutrophil infiltration in *Abcc6* KO animals following PBS or clodronate liposomes treatment. (**A**) Representative flow cytometry plots and gating strategy for identifying macrophages and neutrophils in cryo-injured *Abcc6* KO mouse hearts treated with PBS or clodronate liposomes (green box: neutrophils; red box: macrophages). (**B–C**) Quantification of (**B**) macrophages and (**C**) neutrophils showing significantly reduced infiltration in *Abcc6* KO animals treated with clodronate liposomes. (n = 6 per group; mean ± SD.; ***p < 0.001, PBS vs. clodronate liposomes treatment; two-tailed unpaired t-test.)

Figure S12

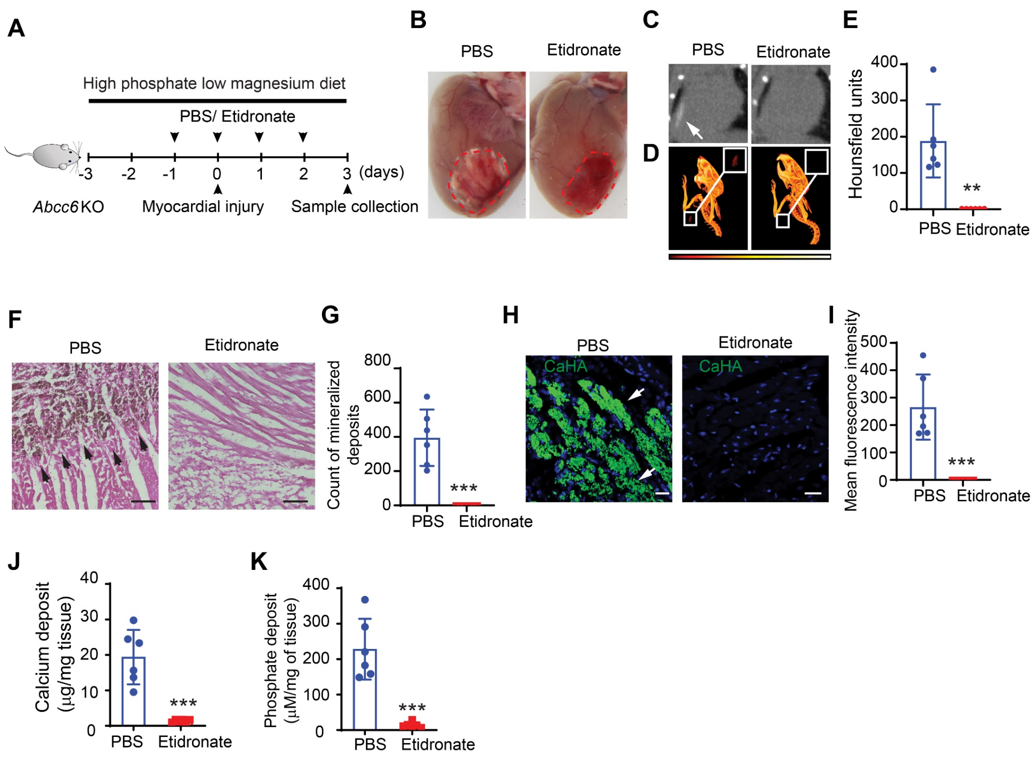

Figure S12. Etidronate inhibits cardiac calcification in animals deficient in *Abcc6*.

(**A**) Experimental design of administration of etidronate in *Abcc6* KO animals to determine effects on cardiac calcification. (**B**) Gross picture of the heart, 3 days post cryo-injury, in *Abcc6* KO animals that received PBS or etidronate (red dotted circles indicate the calcific deposits in the area of injury, arrows indicate the calcific lesion). Note that the animals that received etidronate did not show any mineralization. (**C**) CT scan and (**D**) 3D rendering and (**E**) quantitative analysis of calcium content in scar tissue. (**F-G**) Histological staining of cryo-injured myocardium with (**F**) Von Kossa staining and (**G**) corresponding quantitative analysis showing calcium deposits. Scale bar: 100 µm. (**H**) Immunostaining for hydroxyapatite and (**I**) corresponding quantitative analysis. Scale bar: 50 µm. (**J-K**) Biochemical measurements of myocardial (**J**) calcium and (**K**) phosphate deposits in the injured region. (n=6 per group; mean ± SD.; **p< 0.01, ***p < 0.001. n.s.: not significant. PBS versus etidronate group, P value was calculated by two-tailed unpaired t-test).
