# Supplementary figures and images for: The liver regulates ectopic calcification in Abcc6-deficient models of pseudoxanthoma elasticum
Source: J Clin Invest. 2026 Mar 10;136(9):e193499. doi: 10.1172/JCI193499 (PMC13132379; doi:10.1172/JCI193499)

Figure S9A

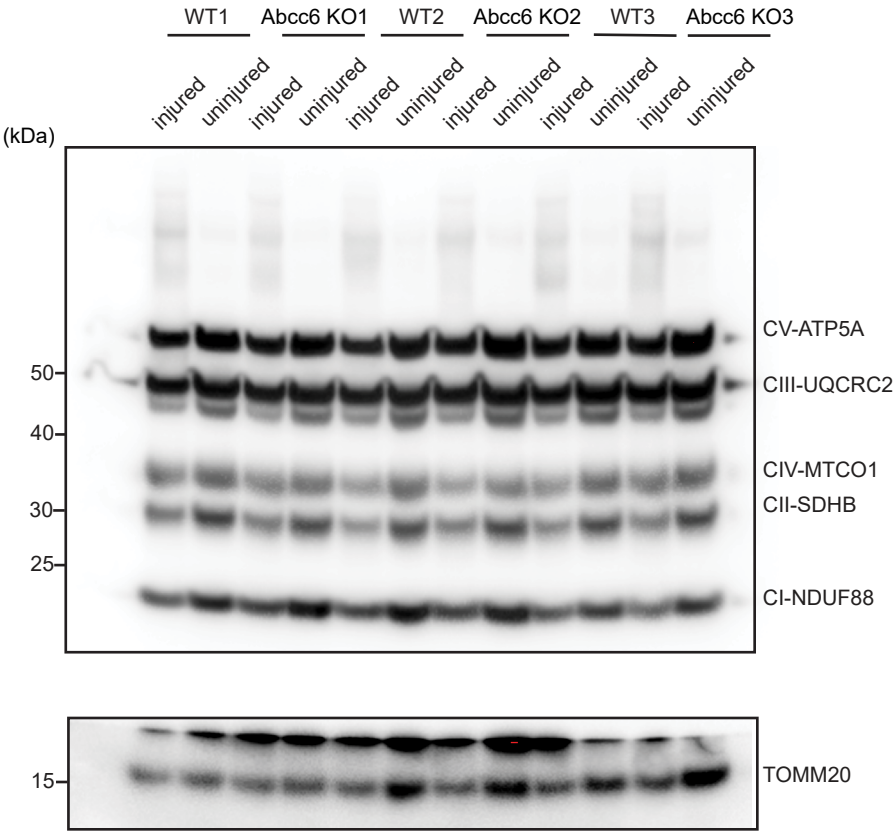

Supplement: Unedited blot and gel images [file jci-136-193499-s149.pdf]
